# Norfloxacin–NSAID Pharmaceutical Salts: Structure–Property Relationships for Enhanced Solubility and Stability

**DOI:** 10.1021/acs.cgd.6c00593

**Published:** 2026-06-11

**Authors:** Jeannette Carolina Belmont-Sánchez, Carolina Alarcón-Payer, Antonio Frontera, Francisco Javier Acebedo-Martínez, Cristóbal Verdugo-Escamilla, Alicia Domínguez-Martín, Duane Choquesillo-Lazarte

**Affiliations:** 1 Laboratorio de Estudios Cristalográficos, 16379 IACT, CSIC, Avda. de las Palmeras 4, Armilla 18100, Spain; 2 Department of Inorganic Chemistry, Faculty of Pharmacy, University of Granada, Granada 18071, Spain; 3 Servicio de Farmacia, Hospital Universitario Virgen de las Nieves, Granada 18014, Spain; 4 Departament de Química, 16745 Universitat de les Illes Balears, Crta. de Valldemossa km 7.5, Palma 07122, Spain; 5 Istituto per lo Studio dei Materiali Nanostrutturati (ISMN)-Consiglio Nazionale Delle Ricerche (CNR), Via P. Gobetti 101, Bologna 40129, Italy

## Abstract

The use of antibiotics is usually associated with mucosal damage and antimicrobial resistance due to inefficient dosing. This issue is particularly relevant for norfloxacin (NOR), whose amphoteric nature leads to strongly pH-dependent solubility and low oral bioavailability. To overcome these limitations, this work presents three novel drug–drug pharmaceutical salts of NOR with the anti-inflammatory drugs ketoprofen (KET), dexketoprofen (DKT), and niflumic acid (NIF). Salts were synthesized by mechanochemical and slurry strategies and fully characterized using thermal, spectroscopic, and X-ray diffraction techniques. The crystal structure, along with DFT and QTAIM computational analyses, showed that salts are governed by tetrameric assembly formation. However, while NOR–KET and NOR–DKT are stabilized by robust charge-assisted hydrogen bonds, NOR–NIF packing arrangement is dominated by weaker hydrogen bonds together with π–π and CF_3_···π interactions. These supramolecular differences were directly reflected in the pharmaceutical performance. The salts suppressed the rapid hydration of NOR under aqueous environments and showed improved stability at physiological pH. Moreover, NOR–KET and NOR–DKT significantly enhanced the solubility of both NOR and the corresponding NSAIDs in PBS at pH 6.8, while all salts provided a more controlled NOR dissolution profile under acidic conditions. These findings demonstrate that salt formation is an effective strategy to overcome the solubility and stability limitations in NOR and NSAIDs, offering a framework for improving the performance not only of NOR but also of other APIs facing similar challenges.

## Graphical abstract

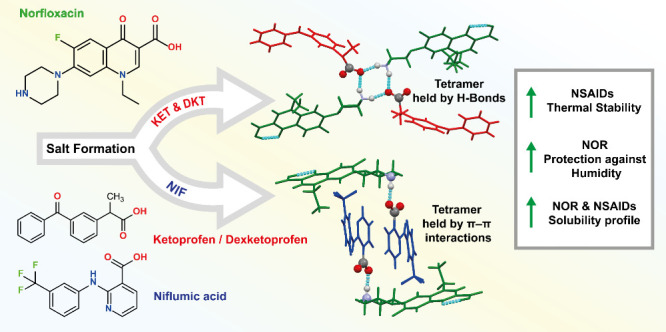

## 1 Introduction

Over the last few decades the World Health Organization has warned about the continuous rise in the incidence of urinary and gastrointestinal infections,^1–3^ mainly driven by the indiscriminate use of antibiotics and the emergence of antimicrobial-resistant bacteria.^4–6^ This global health challenge highlights the urgent need for strict control and rational use of antibiotics.

Besides antibiotic regulation, a major issue associated with many active pharmaceutical ingredients (APIs) is their poor oral bioavailability,^7^ which often necessitates the administration of high doses to achieve therapeutic plasma concentrations.^8,9^ Antibiotics such as norfloxacin (**NOR**, Figure 1) exemplify this problem. **NOR** is a fluoroquinolone antibiotic with potent activity against Gram-negative bacteria, widely prescribed for urinary, renal, and gastrointestinal infections.^10,11^ Despite its efficacy, **NOR** belongs to Class II in the Biopharmaceutics Classification System, characterized by low solubility but high permeability. This results in a low oral bioavailability (30–40%), primarily due to its amphoteric nature and limited solubility under physiological pH conditions.^12^ Although **NOR** is highly soluble in acidic media and readily dissolves in the stomach, its absorption predominantly occurs in the intestine (duodenum and jejunum), where the near-neutral pH reduces its solubility, limiting intestinal absorption and requiring higher doses to achieve therapeutic levels, which can be problematic in acute infections that require a rapid therapeutic response.^13^ Furthermore, precipitation of **NOR** in the intestinal lumen increases its local concentration, potentially irritating the intestinal mucosa, disrupting the microbiota, and contributing to the development of bacterial resistance.^14,15^

**Figure 1:**
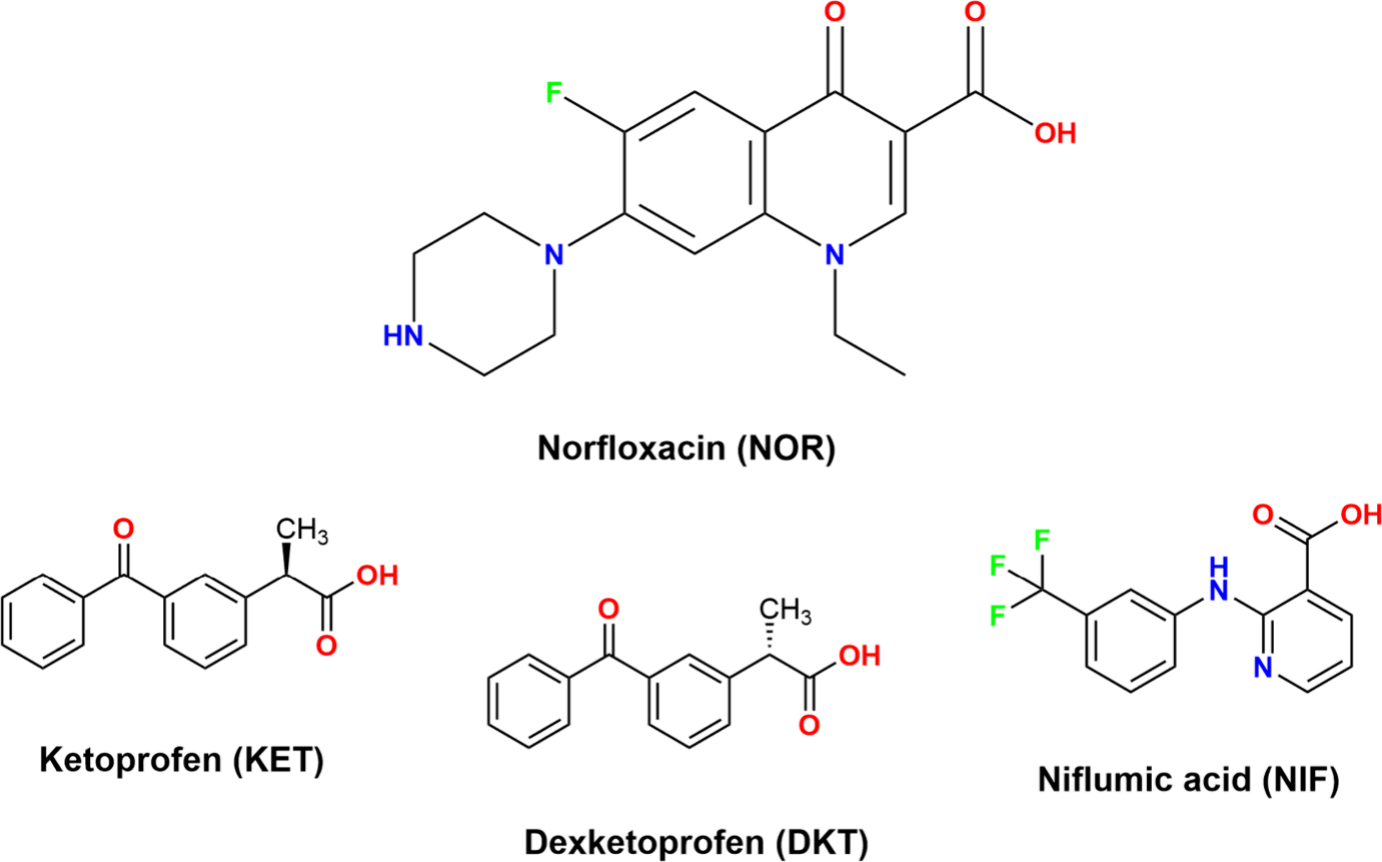
Molecular diagrams of norfloxacin (**NOR**), ketoprofen (**KET**), dexketoprofen (**DKT**) and niflumic acid (**NIF).**.

Improving **NOR** solubility at physiological pH therefore represents a promising strategy to enhance its oral bioavailability and mitigate pharmaceutical drawbacks. In this context, crystal engineering, particularly the development of pharmaceutical multicomponent materials (PMMs), has emerged as an effective and versatile approach.^16,17^ These systems are crystalline solids composed of an API and one or more coformer molecules, that are stabilized by noncovalent interactions, including hydrogen bonding, van der Waals forces, and π–π stacking interactions.^18,19^ The resulting crystal structure allows the modulation of key physicochemical properties, including solubility, thermal stability, and mechanical behavior without altering the molecular structure of the individual components, thereby preserving their pharmacological activity.^20–24^

One step forward in this strategy involves the use of a second API as coformer, resulting in dual-drug multicomponent systems with potential therapeutic synergy.^25–28^ In the case of antibiotics, nonsteroidal anti-inflammatory drugs (**NSAIDs**), such as ketoprofen (**KET**), dexketoprofen (**DKT**), or niflumic acid (**NIF**) (Figure 1) represent promising coformers, given their frequent coadministration in clinical settings where infectious diseases are accompanied by inflammation.^29–31^

In fact, **NSAIDs** are among the most widely consumed drugs for the management of acute and chronic pain, the attenuation of inflammatory responses, and the reduction of fever.^32,33^ Despite their extensive prescription, **NSAIDs** are also associated with a wide range of dose-dependent side effects, many of which are related to their limited aqueous solubility.^34^ Consequently, PMMs offer a rational strategy to simultaneously modulate the physicochemical properties of both **NOR** and **NSAIDs** while combining antibacterial and anti-inflammatory properties within a single crystalline phase.

In this work, we present the synthesis and comprehensive characterization of three novel **NOR–NSAID** salts. A detailed study of the crystal structures and the noncovalent interactions within the crystal lattice is conducted to understand the physicochemical properties exhibited by the developed materials. The structure–property relationships established in this study provide a solid basis for the rational design of PMMs with improved solubility under physiological pH conditions, thereby promoting intestinal absorption and oral bioavailability, as well as potential synergistic therapeutic effects arising from the coadministration of both drugs within a single solid-state material.

## 2 Experimental Section

**NOR**, **DKT**, **KET**, **NIF**, and all solvents were purchased from Sigma-Aldrich (purity >98%, Sigma-Aldrich, St. Louis, MO, USA) and were used directly as received.

### 2.1 NOR–NSAIDs Salt Synthesis

The mechanochemical synthesis of **NOR–KET** and **NOR–DKT** salts was carried out via liquid-assisted grinding (LAG) in a RETSCH MM2000 ball mill, operating at 25 Hz frequency for 30 min. For this purpose, equimolar mixtures of **NOR** (0.5 mmol, 159.66 mg) and the corresponding **NSAID** (0.5 mmol, 127.14 mg) were weighed and placed into stainless steel jars of 20 mL of volume, along with two stainless steel balls of 7 mm diameter and 100 μL of methanol (MET). **NOR–NIF** was obtained via slurry operations, in which equimolar mixtures of **NOR** (0.5 mmol, 159.66 mg) and **NIF** (0.5 mmol, 141.11 mg) were weighed and placed in 5 mL vials along with 1 mL of MET and one magnetic stirrer. After 72 h of agitation, the suspension was centrifuged and the pellet air-dried. All solid products obtained were analyzed by PXRD to determine the formation of the novel materials.

Single crystals of the phases were obtained by slow solvent evaporation at room temperature, using saturated solutions of the material obtained from the synthesis in ethyl acetate (ETA). Suitable crystals for SCXRD appeared after 2 days of slow solvent evaporation at room temperature.

### 2.4 Characterization Techniques

#### 2.4.1 X-ray Diffraction Analysis

Powder X-ray diffraction (PXRD) analysis was carried out at room temperature on a Bruker D8 Advance Vαrio diffractometer (Bruker-AXS, Karlsruhe, Germany) equipped with a LYNXEYE detector and CuKα_1_ radiation (1.5406 Å). Generator operating conditions were 40 kV and 40 mA and measurements were performed in transmission geometry between Mylar foils with the beam focused in the detector. The diffractograms were obtained over an angular range of 5–40° (2θ) with a step size of 0.02° (2θ) and a constant counting time of 5 s per step.

Suitable crystals or Single-Crystal X-ray diffraction (SCXRD) were prepared under inert conditions using perfluoropolyether as protective oil for manipulation. Crystals were placed on MiTeGen Micromounts and data were collected with a Bruker D8 Venture diffractometer (Bruker-AXS, Karlsruhe, Germany) with graphite monochromated CuKα radiation (λ = 1.54178 Å). Integration and scaling of the intensity data were accomplished using the APEX4 suite.^35^ The structures were solved by intrinsic phasing using the ShelXT program,^36^ which revealed the position of all non-hydrogen atoms. These atoms were refined on F^2^ by a full-matrix least-squares procedure using the anisotropic displacement parameter.^37^ All hydrogen atoms were located in difference Fourier maps and included as fixed contributions riding on attached atoms with isotropic thermal displacement parameters 1.2 or 1.5 times those of the respective atom. The Olex2 ^38^ software was used as a graphical interface. Intermolecular interactions were calculated using PLATON.^39^ Molecular graphics were generated using Olex2 ^38^ and Mercury.^40^ The crystallographic data for the reported structures were deposited with the Cambridge Crystallographic Data Center as supplementary publication no. CCDC 2550127, 2550128 and 2550129.

#### 2.4.2 Computational Methods

All quantum chemical calculations were computed either Gaussian-16.^41^ The initial geometries for the molecular assemblies (dimers, trimers, and tetramers) were taken directly from their experimental SCXRD structures. This approach was chosen to analyze supramolecular interactions as they exist in the solid state, thereby preserving the subtle balance of forces and molecular conformations present in the crystalline environment, rather than using optimized gas-phase geometries.

Interaction energies were computed using Density Functional Theory (DFT) with the PBE0-D3 ^43^/def2-TZVP^44^ model chemistry.

The PBE0 hybrid functional provides a balanced description of both covalent and noncovalent interactions.^42^ Grimme’s D3 empirical dispersion correction was included to accurately describe the critical long-range dispersion forces,^43^ and the def2-TZVP basis set was used to ensure high accuracy.^44^ All interaction energies were corrected for Basis Set Superposition Error (BSSE) using the standard counterpoise method.^45^

Topological analysis of the electron density was conducted using the Quantum Theory of Atoms in Molecules (QTAIM)^46^ and the Non-Covalent Interaction (NCI) index,^47,48^ as implemented in the AIMAll program package.^49^ The QTAIM analysis identified bond critical points (BCPs) and bond paths for intermolecular contacts, with hydrogen bond strengths estimated using the local energy density predictor, (EH-bond ≈ 0.5 × V).^50^ The NCI analysis provided visual confirmation of these interactions through reduced density gradient (RDG) isosurfaces. Finally, Molecular Electrostatic Potential (MEP) surfaces were generated using GaussView^51^ to assess intrinsic electron donor–acceptor propensities and identify favorable sites for hydrogen bonding.

#### 2.4.3 Fourier Transform Infrared Spectroscopy (FT-IR)

Fourier transform infrared (FTIR) spectroscopic measurements were performed on a Bruker Tensor 27 FT-IR instrument (Bruker Corporation, Billerica, MA, USA) equipped with a single-reflection diamond crystal platinum ATR unit and an OPUS data collection program. The scanning range was 4000–400 cm^–1^, with a resolution of 4 cm^–1^.

#### 2.4.4 Thermal Analysis

Simultaneous differential scanning calorimetry (DSC) and thermogravimetric analysis (TGA) were performed in a NETZSCH STA 449 F5 calorimeter (NETZSCH Group, Germany). Experimental conditions: alumina (Al_2_O_3_) crucibles of 85 μL volume, atmosphere of dry nitrogen with 250 mL/min flow rate, and heating rates of 5 °C/min ranging from 25 to 300 °C. The calorimeter was calibrated with indium of 99.99% purity (m.p.: 156.4 °C; DH: 28.14 J/g).

#### 2.4.5 Stability Studies

Stability in aqueous solution of the APIs and their derived PMMs was evaluated through slurry experiments, in which, an excess of the solid was added to 10 mL of buffer PBS pH 6.8 or of KCl pH 1.2. After 24 h of stirring at 25 °C in sealed vials, the solids were collected, filtered, dried at 35 °C and further analyzed by PXRD.

The stability of the samples was also assessed under accelerated aging conditions (40 °C and 75% RH), placing powder samples in a Memmert HPP110 climate chamber (Memmert, Schwabach, Germany) up to 3 months. The samples were analyzed by PXRD periodically to evaluate possible phase transformation during the time of the experiment.

#### 2.4.6 Powder Dissolution Profile

Powder dissolution profile studies were undertaken following the shake-flask method^52^ in KCl pH 1.2 and PBS pH 6.8. Saturated solutions of **NOR**, **NSAIDs** and **NOR–NSAIDs** salts, were prepared by adding an excess of solid to 10 mL of each buffer. During 24 h of stirring at 25 °C, aliquots of the solutions were filtered through 0.22 μm syringe filters, diluted to achieve a measurable concentration and analyzed via high-performance liquid chromatography (HPLC).

HPLC studies were performed using a HPLC Agilent 1200 series and a C18 column (4.6 mm × 150 mm, 4 μm particle size, Phenomenex) set at 40 °C. The mobile phase consisted of (A) PBS, 0.04 M, pH 2.5 and (B) Acetonitrile (purity >99%, Sigma-Aldrich, St Louis MO, USA). The initial mobile-phase composition was 85% A and 15% B, which was held isocratically for 4.4 min. A linear gradient was then applied, increasing solvent B from 15% to approximately 70% between 4.4 and 5.5 min. This composition was maintained isocratically until the end of the run (11 min). Flow rate was adjusted to 1 mL/min and 10 μL of sample was injected. The solubility (expressed in mg/mL) was evaluated at 254, 278, and 289 nm maximums of absorbance of KET/DKT, NOR and NIF.

## 3 Results and Discussion

### 3.1 NOR–NSAID Salts Synthesis

Initial attempts to obtain the **NOR**–**NSAID** phases were carried out using neat grinding to prioritize organic solvent-free synthesis routes. However, as shown in Figure S1, the PXRD patterns revealed only physical mixtures of **NOR** and the respective **NSAID**. To induce the formation of novel solid phases, LAG was employed. Mechanochemical approaches, and particularly LAG, have become widely used methodologies for the rapid, efficient, and sustainable preparation of pharmaceutical salts and cocrystals. Compared with conventional solution-based strategies, LAG drastically reduces solvent consumption, shortens reaction times, and often improves reaction selectivity and reproducibility.^53^ In this sense, the use of LAG is closely aligned with the principles of green chemistry, minimizing waste generation and e environmental impact. Moreover, mechanochemical strategies have been recognized as sustainable chemical processes aligned with the principles of green chemistry and the objectives of the United Nations Sustainable Development Goals.^54^

LAG reactions were first performed using water as liquid additive to maintain *green* processing conditions. Nevertheless, as shown in Figure S1 these reactions predominantly yielded a mixture of unreacted **NSAID** and an unknown phase, most likely a hydrated form of **NOR**, due to the already reported disposition of **NOR** to form hydrates. However, none of the NOR hydrates reported in the CCDC matches the phase observed in the aqueous LAG.^55,56^

At this point, different organic solvents were screened. For **NOR–KET** and **NOR–DKT**, positive results were achieved using MET. Figure 2 shows the PXRD patterns of the reaction products demonstrating the formation of new phases, with no signals of the parent components.

**Figure 2:**
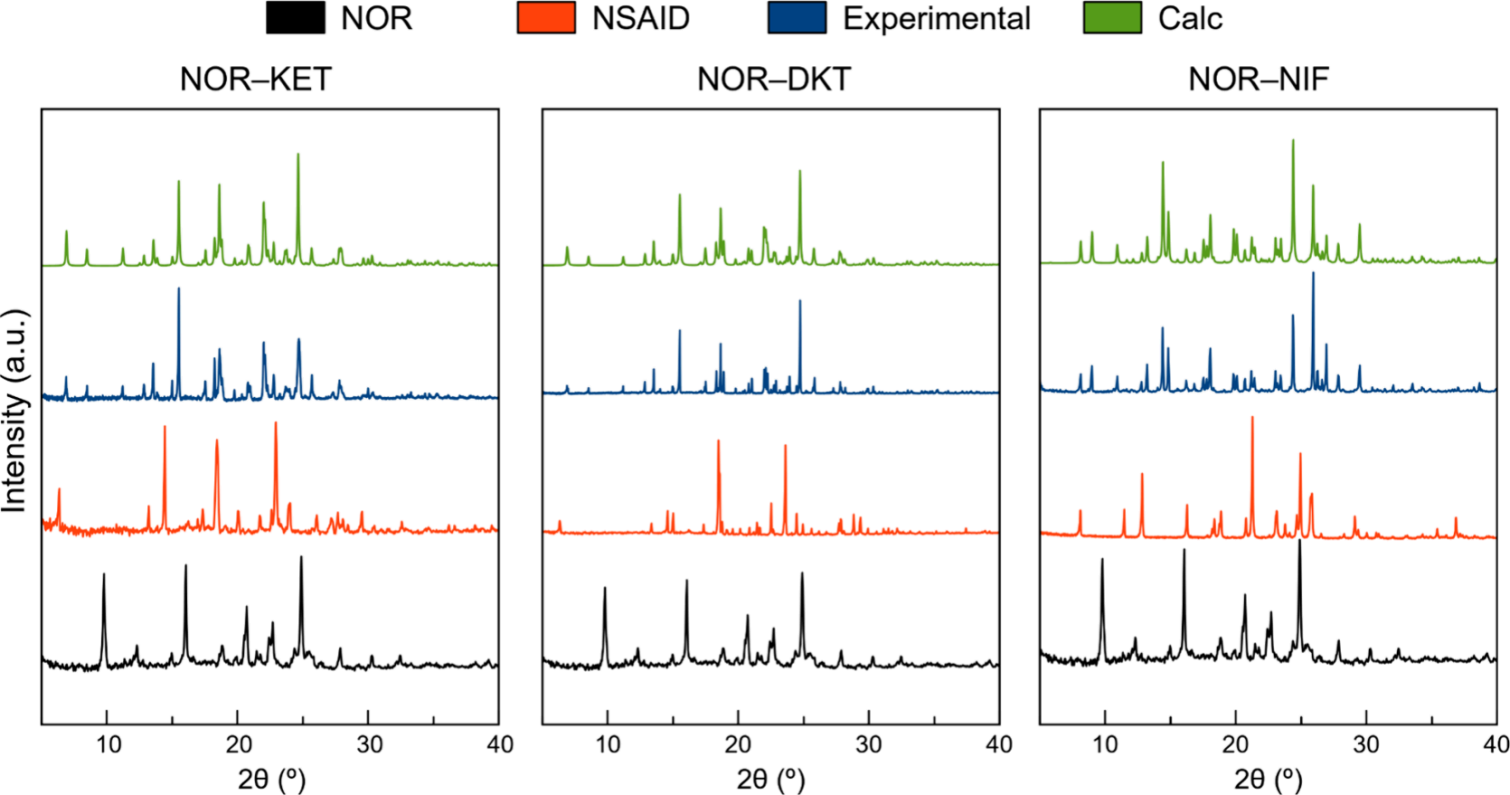
PXRD patterns of the products obtained from LAG (**NOR–KET** and **NOR–DKT**) and slurry (**NOR–NIF**) reactions compared with the parent APIs and the calculated (Calc) PXRD pattern of the phases derived from their corresponding crystal structures.

In contrast, LAG of **NOR** with **NIF** using MET and a variety of organic solvents produced physical mixtures of the components or poorly crystalline solids that could not be isolated (see Figure S2a). Poor crystalline products indicated amorphization, attributed to an excess of energy during the LAG reaction. For this reason, a less energetic and more thermodynamic approach was adopted: slurry crystallization. In this method, the components were suspended in a small volume of MET (to minimize dissolution of the APIs) and allowed to equilibrate under continuous stirring. Experiments were performed at 24, 48, 72, and 96 h (Figure S2b), but only after 72 h, a novel crystalline phase was obtained, without any signals of the parent APIs (Figure 2. This indicates that **NOR–NIF** system does not benefit from more energetic techniques such as LAG. Furthermore, the absence of product formation at 24 and 48 h suggests that the phase emerges only after a prolonged equilibration period, indicating that the higher volume of solvent used in slurry (compared to LAG) is not the main reason for the formation, but rather the time of equilibrium and reaction.

At this stage, PXRD alone was insufficient to distinguish whether the materials corresponded to a new salt, cocrystal, or a derived phase from an API (such as solvates or polymorphs). For this reason, FT-IR spectroscopy was used as complementary technique. Shifts in characteristic bands of functional groups that are involved in supramolecular interactions supported the formation of PMMs and provide information about the nature of the materials. Figure S3 shows the FT-IR spectra of the **NOR**–**NSAID** products, while the most relevant FT-IR bands are summarized in Table 1 for clarity.

**Table 1:** Main FT-IR Bands (cm^–1^) Ascribed to the Principal Functional Groups of **NOR** and **NSAIDs**, along with the Shift of the Bands in the Respective PMMs

compound	υ(N–H)	υ(O–H)_acid_	υ(C=O)_acid_	**υ**(**C–F)**
**NOR**	3600–3200	3090–2422	1727	1102
**KET**		3216–2328	1696	
**DKT**		3408–2560	1730	
**NIF**	3391–3241	3136–2387	1666	1109
**NOR–KET**	3060	2353	1709	1139
**NOR–DKT**	3055	2350	1714	1134
**NOR–NIF**	3055	2356	1707	1105

For **KET**, the υ­(O–H) stretching is observed between 3216–2328 cm^–1^, while the υ­(C = O) band of the carboxylic acid appears at 1696–1655 cm^–1^. In **DKT**, the υ­(O–H) band is detected at 3408–2560 cm^–1^ and the υ­(C = O) stretching associated with the acid group at 1730–1650 cm^–1^. Similarly, in **NIF**, the υ­(O–H) stretching lies within 3136–2387 cm^–1^, and the carboxylic υ­(C = O) band appears at 1666 cm^–1^. In **NOR–KET**, **NOR–DKT**, and **NOR–NIF**, the carboxylic υ­(C = O) bands of the **NSAIDs** appear at lower wavenumbers compared to the pure APIs, being observed at 1709 cm^–1^, 1714 cm^–1^, and 1707 cm^–1^, respectively. These shifts are consistent with the deprotonation of the carboxylic acid and the formation of a carboxylate stabilized through interaction with **NOR**. The disappearance or marked alteration of the υ­(O–H) stretching bands in the resulting solids aligns with the loss of the acidic proton.

Meanwhile, **NOR** exhibits the characteristic signals of amine and amide functionalities, with the υ­(N–H) stretching at 3600–3200 cm^–1^, the amide υ­(C = O) at 1620 cm^–1^, and the υ­(C–N) stretching around 1250 cm^–1^. The amine and amide groups of **NOR** also exhibit changes after interaction with the **NSAIDs**. The υ­(N–H) stretching band, originally located at 3600–3200 cm^–1^, appears at 3060 cm^–1^ in **NOR–KET** and at 3055 cm^–1^ in both **NOR–DKT** and **NOR–NIF**. Likewise, the υ­(C–N) stretching band of **NOR**, initially at 1250 cm^–1^, shifts to 1273 cm^–1^ in **NOR–KET**, 1271 cm^–1^ in **NOR–DKT**, and 1273 cm^–1^ in **NOR–NIF**. These results point to the formation of pharmaceutical salts, stabilized by NH_2_^+^···OOC^–^hydrogen bonds.

### 3.2 Crystal Structure Analysis

To corroborate PXRD and FT-IR results, single crystals were grown and subjected to SCXRD analysis. The resulting crystal structures confirmed that all **NOR**–**NSAID** are true pharmaceutical salts formed between protonated **NOR** (**NOR** ^+^) and the deprotonated **NSAID** (**KET** ^–^, **DKT** ^–^ or **NIF** ^–^). In addition, the crystal structures allowed the calculation of PXRD patterns, which, upon comparison with the experimental ones (Figure 2), verified the purity and reproducibility of the synthesis reactions.

**NOR–KET** and **NOR–DKT** display closely related crystal structures due to their chemical relationship. **KET** is a racemic mixture containing both R- and S-enantiomers, whereas **DKT** is the enantiopure S-form. This difference is relevant for understanding the crystallographic description of both salts, particularly regarding the content of the asymmetric unit and the role of symmetry operations within the crystal structure.

**NOR–KET** crystallizes in the triclinic system, *P*1̅ space group, with a 1:1 stoichiometric ratio (the asymmetric unit is composed of one **NOR** ^+^ and one **KET** ^–^). The ionic pair is stabilized by a strong charge-assisted hydrogen bond (CAHB) between the deprotonated carboxylate group of **KET** ^**–**^ and the piperazine ammonium group of **NOR** ^+^. This main NH_2_^+^···OOC^–^ interaction is described by an *D*_1_^1^(2) graph set (Figure 3a). In the asymmetric unit, an additional intramolecular hydrogen bond within **NOR** ^+^ allows to improve the cohesion of the ionic pair. Because **NOR–KET** crystallizes in the centrosymmetric *P*1̅ space group, the inversion center generates a second symmetric ionic pair containing the opposite **KET** enantiomer. These two ionic pairs assemble through equivalent N–H···OOC interactions to generate a tetrameric unit, resulting in a ring-type hydrogen-bond framework described by an *R*_4_^2^(8) graph set (Figure 3b). Within this synthon, each **NOR** ^+^ contributes the two hydrogen atoms of its protonated piperazine, and each **KET** ^–^ uses one oxygen atom of its carboxylate group to form two N–H···OOC hydrogen bonds. To generate the three-dimensional structure, tetramers are further connected through reciprocal C–H···O interactions between adjacent **NOR** ^+^ molecules, generating zigzag chains of **NOR** ^**+**^ cations that extend along the *ac* axis, while **KET** ^**–**^ anions remain laterally disposed (Figure 3b).

**Figure 3:**
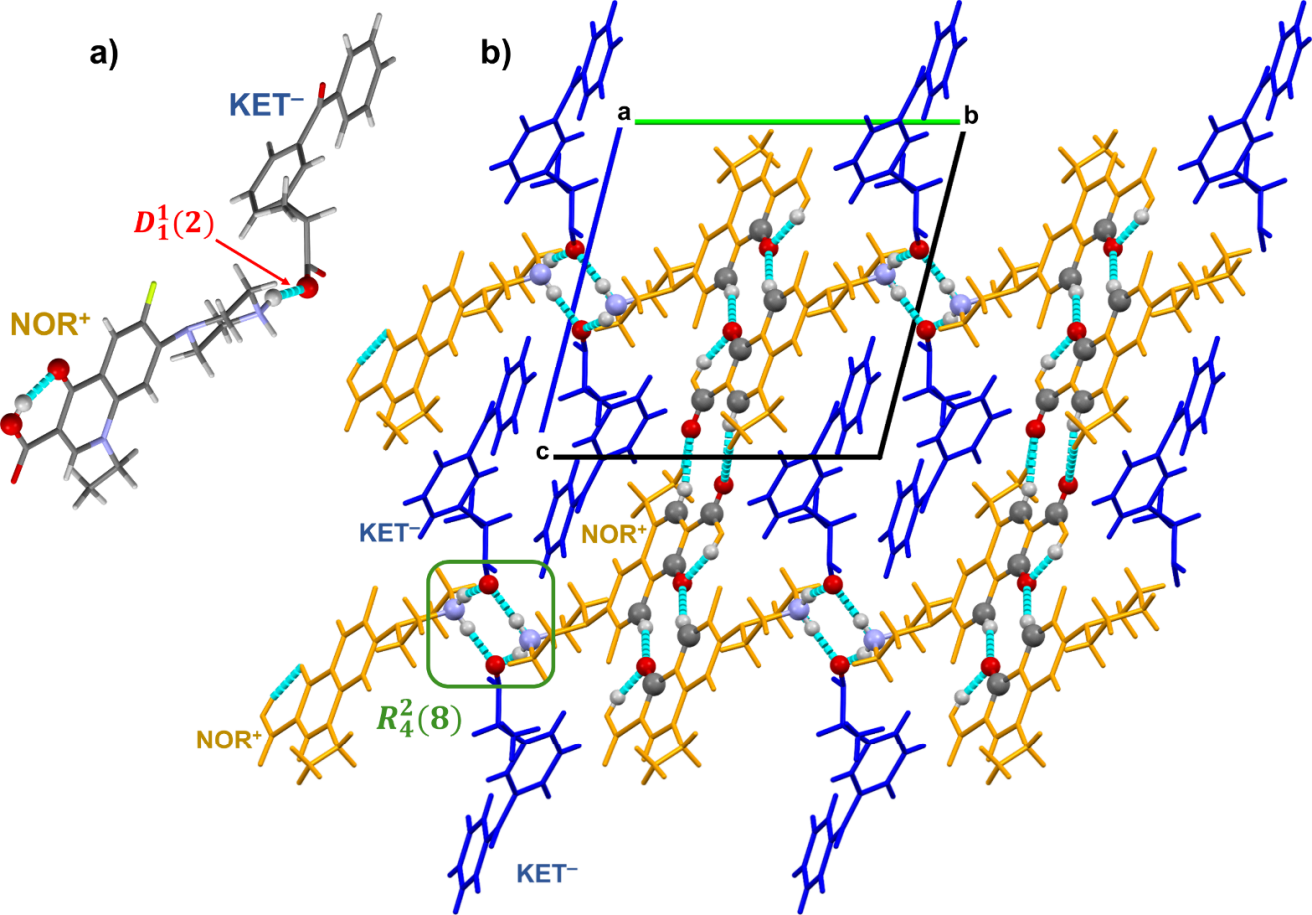
Structural features of **NOR–KET**. a) Major *D*_1_^1^(2) graph set established between **NOR** ^+^ and **KET** ^**–**^ in the asymmetric unit. b) Supramolecular organization of **NOR** ^+^ (orange) and **KET** ^–^ (blue). Green boxes highlight the connection of tetrameric units by *R*_4_^2^(8) graph sets. Atoms involved in the interactions are presented as balls, while the rest are presented as sticks.

**NOR–DKT** also crystallizes in the triclinic system but in the noncentrosymmetric P1 space group. Consequently, the asymmetric unit contains two independent **NOR** ^+^ cations and two **DKT** ^–^ anions, all involving the S-enantiomer (**DKT**). These two ionic pairs are directly present in the asymmetric unit and assemble into a tetrameric architecture (*R*_4_^2^(8)) similar to that observed in **NOR–KET**. Therefore, although the Z values presented in Table 2 differ between the two salts, both unit cells contain two **NOR–NSAID** ionic pairs. The three-dimensional organization in **NOR–DKT** follows the same structural pattern described for **NOR–KET**. The close structural similarity between both salts is supported by molecular overlay analysis (Figure 4), which gives root-mean-square deviation (RMSD) values of 0.027 Å for **NOR** ^+^ and 0.1098 Å for the **NSAIDs** ^–^, indicating only minor deviations in molecular conformation, arising from the use of enantiopure **DKT**. This high degree of similarity is also reflected in the comparable unit-cell parameters summarized in Table 2.

**Figure 4:**
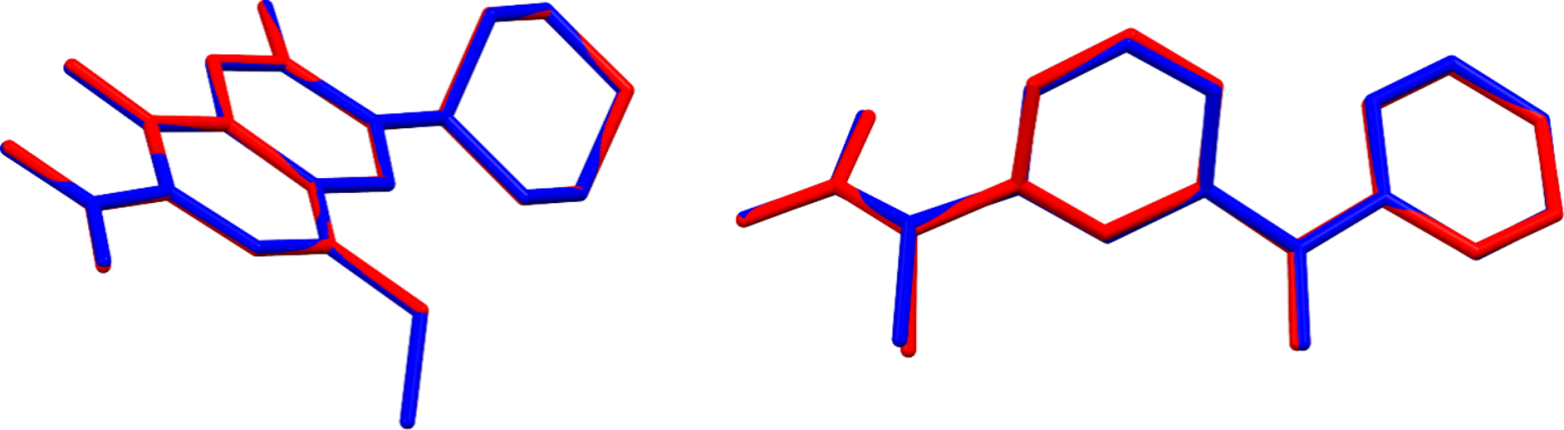
Overlap between two independent molecules of **NOR** ^+^ and **NSAIDs** ^**–**^, in the crystal structures of **NOR–KET** (blue) and **NOR–DKT** (red). Hydrogen atoms are omitted for clarity.

**Table 2:** Crystallographic Data of **NOR–KET**, **NOR–DKT** and **NOR–NIF**

compound name	NOR–KET	NOR–DKT	NOR–NIF
CCDC number	2550127	2550128	2550129
crystal system	triclinic	triclinic	triclinic
space group	*P*1̅(2)	*P*1(1)	*P*1̅(2)
*a* [Å]	8.3289(2)	8.3654(13)	8.056(4)
*b* [Å]	13.1499(2)	13.068(2)	11.090(6)
*c* [Å]	13.7364(2)	13.747(3)	15.503(7)
α [°]	103.4850(10)	103.009(9)	101.59(2)
β [°]	106.0490(10)	106.138(7)	92.70(2)
γ [°]	91.3220(10)	91.476(6)	93.43(2)
volume [Å^3^]	1399.79(5)	1400.3(4)	1351.9(11)
*Z*	2	1	2
ρ_calc_[g cm^–3^]	1.361	1.360	1.478
*F*(000)	604	604	624
reflections collected	24233	37384	20807
independent reflections	4912	9238	4727
	*R*_int_ = 0.0393	*R*_int_ = 0.0552	*R*_int_ = 0.0565
data/restraints/parameters	4912/0/402	9238/3/774	4727/78/419
goodness-of-fit on *F* ^2^	1.049	1.068	1.052
final *R* indexes	*R*_1_ = 0.0556	*R*_1_ = 0.0565	*R*_1_ = 0.0483
[*I* ≥ 2σ(*I*)]	w*R*_2_ = 0.1415	w*R*_2_ = 0.1435	w*R*_2_ = 0.1247

Similarly, **NOR–NIF** crystallizes in the triclinic system, *P*1̅ space group with an asymmetric unit containing one **NOR** ^+^ and one **NIF** ^–^. The ions are connected through the same CAHB, NH_2_^+^···OOC^–^ (*D*_1_^1^(2) graph set) observed in the previous salts. However, in this case the ionic pair is oriented, creating a right angle of almost 90 ° (Figure 5a). Within the asymmetric unit, two additional intramolecular interactions are observed. In **NOR** ^**+**^, a strong O–H···O hydrogen bond is established between the carboxylic acid hydroxyl group and the adjacent quinolone oxygen atom, allowing the carboxylate group’s orientation to be locked. In **NIF** ^–^, the interaction is an N–H···O H hydrogen bond between the carboxylic acid hydroxyl group and the adjacent nitrogen atom, which is also key to maintaining the planar conformation of the molecule’s core. As a consequence of the centrosymmetric the centrosymmetric *P*1̅ space group, the ionic pair is related to a second **NOR** ^**+**^ **/NIF** ^**–**^ pair by inversion symmetry, which leads to the formation of a tetrameric assembly inside the unit cell (Figure 5b). However, in this particular case, the two ionic pairs are connected via π···π interactions between the aromatic rings of **NIF** ^**+**^. As shown in Figure 5c, these assemblies propagate along the *b* axis through N–H···O interaction between adjacent **NOR** ^+^, generating one-dimensional chains. In this arrangement, **NOR** ^+^ molecules remain approximately parallel to the chain direction, while **NIF** ^–^ anions are disposed nearly perpendicular. Finally, the one-dimensional chains are connected through additional π–π interactions involving NOR^+^ molecules, leading to the final three-dimensional supramolecular structure.

**Figure 5:**
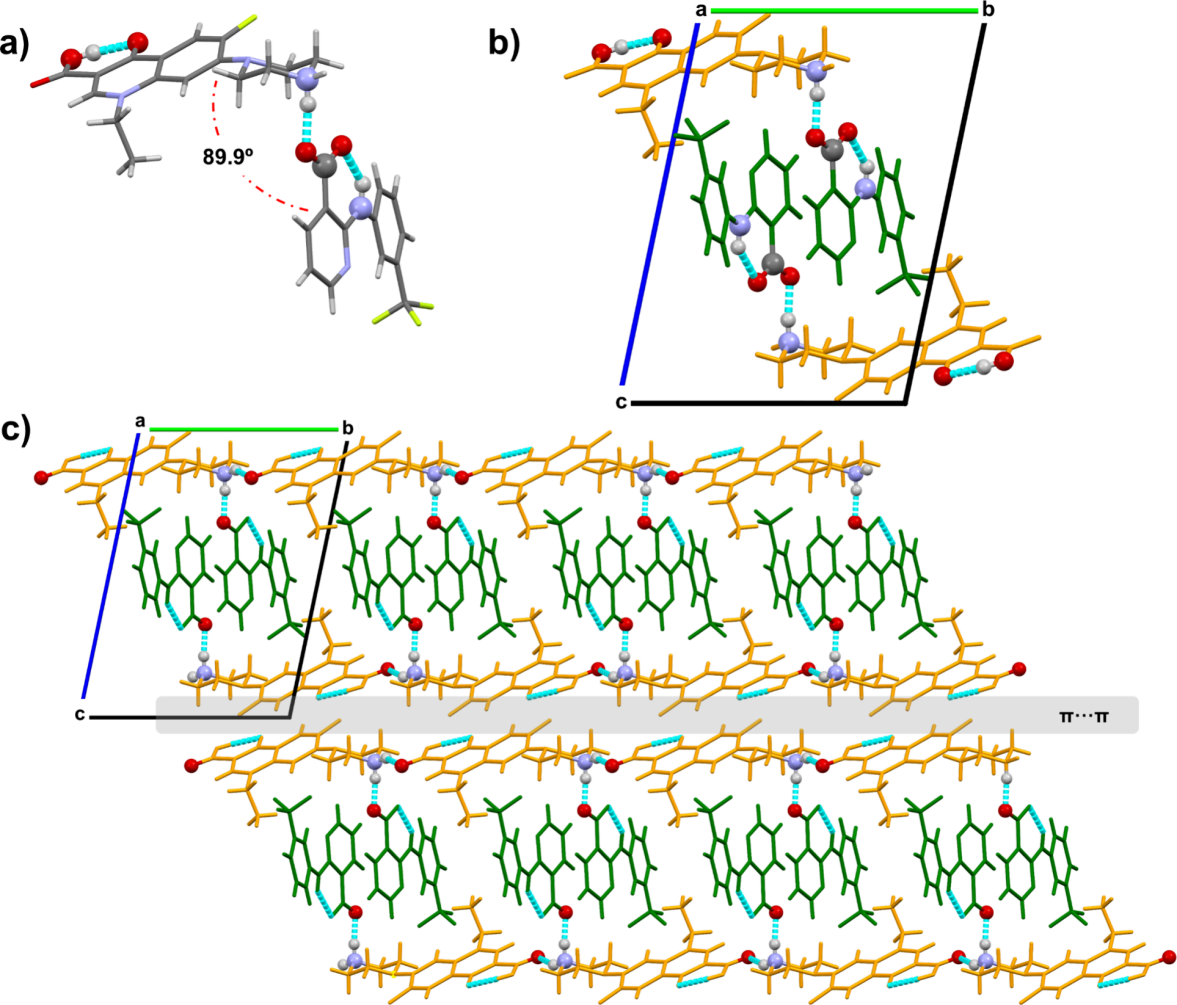
Structural features of **NOR–NIF**. a) Asymmetric unit and the major *D*_1_^1^(2) graph set established between **NOR** ^+^ and **NIF** ^–^. b) Tetrameric formation inside the unit cell. c) Supramolecular organization of **NOR** ^+^ (orange) and **NIF** ^**–**^ (green). Atoms involved in the interactions are presented as balls, while the rest are presented as sticks. Gray box represents π···π stacking interaction areas.

### 3.3 Computational Studies

Following the successful synthesis and SCXRD characterization of the three pharmaceutical salts, we performed a detailed theoretical analysis of the observed solid-state supramolecular architecture.

To understand the intrinsic electron donor–acceptor ability of the coformers and rationalize the observed CAHB preferences, we computed the MEP surfaces for the isolated ionic species at the same level of theory used for the full interaction energy calculations, specifically PBE0-D3/def2-TZVP. The computed MEP surfaces for the **DKT** ^**–**^ (serving also as model for the **KET** ^–^), **NIF** ^–^, and **NOR** ^+^ are shown in Figure 6. The MEP surfaces for **DKT** ^**–**^ and **NIF** ^**–**^, are negative across the entire surface due to the overall negative charge, confirming their nature as electron-rich hydrogen bond acceptors. The most electron-rich regions (MEP minima) are quantified as follows: for **DKT** ^**–**^, the absolute MEP minimum is located at one of the carboxylate oxygen atoms with a value of – 148.0 kcal/mol, with a second significant negative region found at the nearby carbonyl oxygen atom, with a MEP value of – 83.5 kcal/mol. The MEP minimum for **NIF** ^**–**^ is also located at the carboxylate oxygen atom, measuring – 137.8 kcal/mol. Another important electron-rich region is observed at the pyridine nitrogen atom, with a value of – 77.8 kcal/mol. The less negative MEP minimum observed for **NIF** ^**–**^ compared to **DKT** ^**–**^ (−137.8 vs – 148.0 kcal/mol) suggests that **DKT** ^**–**^/**KET** ^–^ anion is intrinsically a stronger hydrogen bond acceptor. This decrease in the MEP value at the **NIF** ^**–**^ carboxylate group is most likely attributable to the already described intramolecular N–H···O hydrogen bond, which partially shields or disperses the negative charge, thus rationalizing why **DKT** ^**–**^/**KET** ^–^ are better CAHB acceptors than **NIF** ^**–**^. The positive MEP values for **NOR** ^**+**^ confirms its strong electron-poor hydrogen bond donor ability, with the MEP maxima located at the hydrogen atoms of the protonated piperazine ring’s ammonium group: the axial N**–**H bond exhibits a slightly larger MEP maximum of 161.3 kcal/mol, and the equatorial N–H bond is slightly less positive, with a value of 160.6 kcal/mol. These data confirm that the ammonium group is the primary site for the formation of N–H···OOC CAHBs, supporting the SCXRD observations. Importantly, the MEP values at the oxygen atoms of the **NOR** ^**+**^ carboxylic acid group are slightly negative, specifically – 15.7 kcal/mol for the carbonyl oxygen and – 6.7 kcal/mol for the hydroxyl oxygen. This residual negative potential provides a rational explanation to the **NOR** ^**+**^⋯**NOR** ^**+**^ chain formation via N–H···O interactions observed in the crystal structure of **NOR–NIF.**

**Figure 6:**
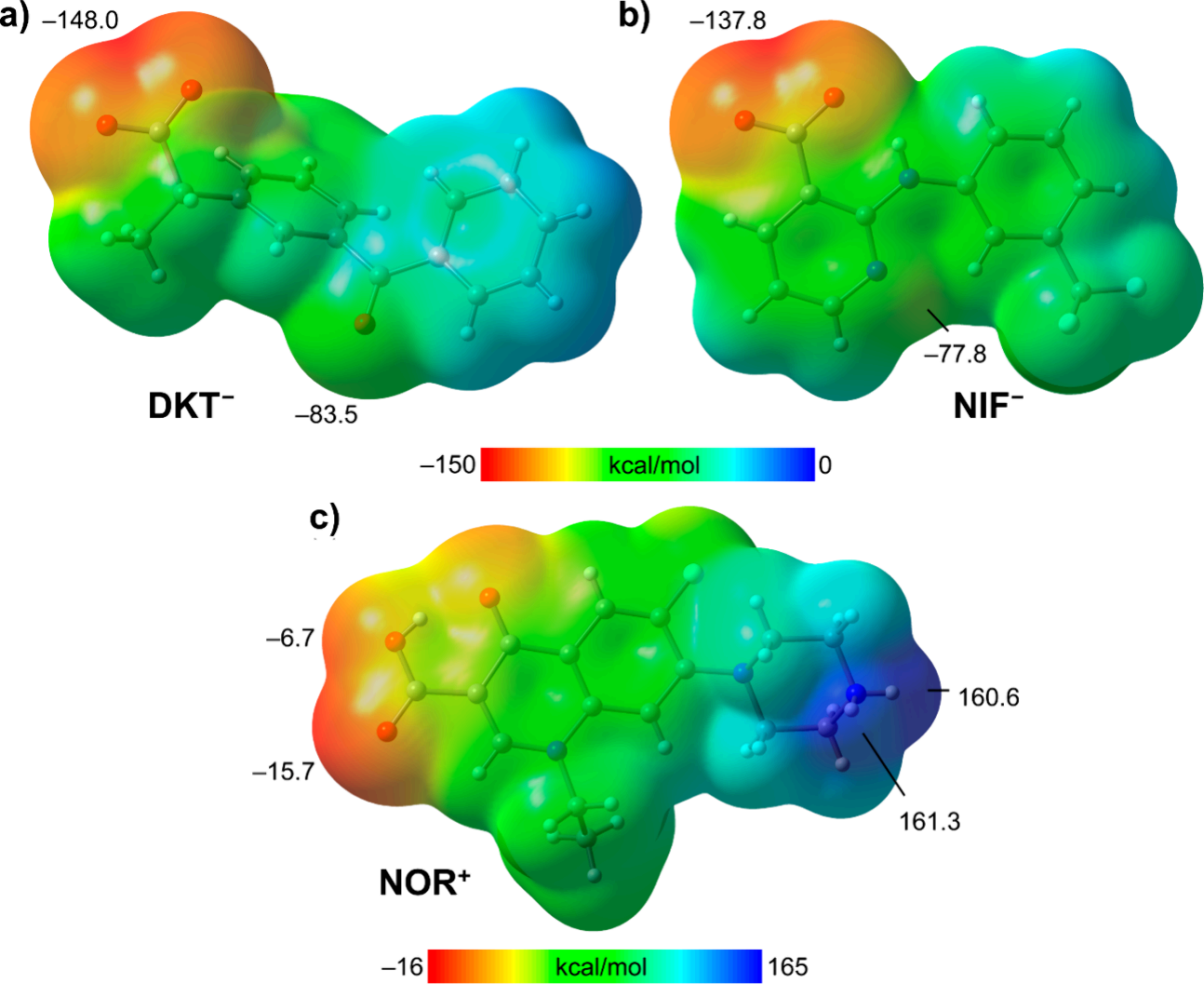
MEP surfaces for the isolated ionic species: a) **DKT** ^**–**^, b) **NIF** ^**–**^, and c) **NOR** ^**+**^. The surfaces map the potential onto the 0.001 au isodensity surface, showing electron-rich regions (red/negative) and electron-poor regions (blue/positive). Key MEP minimum (negative) and maximum (positive) values are labeled, indicating the primary hydrogen bond acceptor sites (carboxylate oxygens in a, b) and donor sites (ammonium N–H groups in c).

The influence of the intramolecular interactions in **NOR** ^**+**^ and **NIF** ^**–**^ leads us to perform a topological analysis of the electronic density, based on the QTAIM. The analysis of the isolated **NOR** ^**+**^ and **NIF** ^**–**^ molecules is visualized in Figure 7. The strength of these interactions, estimated using an energy predictor based on the potential energy density at the bond critical point (E ≈ 0.5 × V, see theoretical methods). In **NOR** ^**+**^ the O–H···O presented an estimated energy of – 12.5 kcal/mol, which is significantly stronger than the C–H···F interaction (−3.9 kcal/mol). Similarly, in **NIF** ^**–**^ the N–H···O contact presented an estimated energy of – 7.9 kcal/mol, while the second C–H···N interaction is – 3.2 kcal/mol. In contrast to **NOR** ^**+**^ and **NIF** ^**–**^, **DKT** ^**–**^ (and thus **KET** ^–^) shows no evidence of intramolecular hydrogen bonds, meaning its conformational flexibility in the gas phase is solely governed by rotational barriers around single bonds.

**Figure 7:**
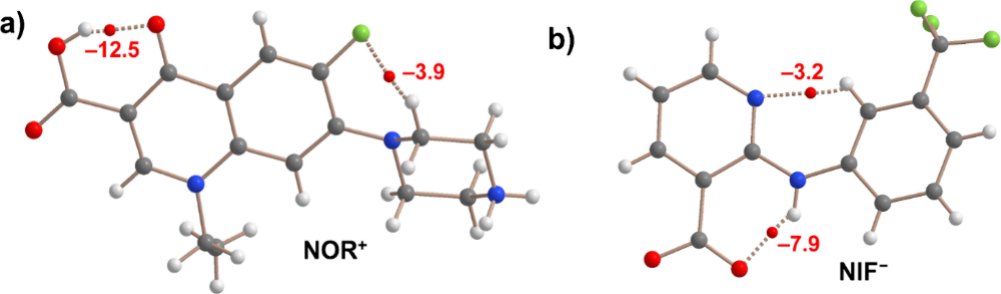
QTAIM analysis for the optimized gas-phase structures of a) **NOR** ^**+**^ and b) **NIF** ^**–**^. Intramolecular interactions are visualized by dashed brown lines (bond paths) and characterized by a small red sphere (BCP). The corresponding stabilization energies for the interactions, are labeled in kcal/mol.

The primary supramolecular synthons observed in the crystal structures were analyzed using a combination of QTAIM and NCIplot methods to quantify the contributions of the individual interactions. Figure 8 illustrates the results for the dominant assemblies in the three salts. SXCRD demonstrated that **NOR–KET** and **NOR–DKT** are governed by the formation of the *R*_4_^2^(8) graph set, which involves two **NOR** ^**+**^ cations and two **KET** ^**–**^/**DKT** ^**–**^ anions, resulting in an eight-membered supramolecular ring. Each N–H···O hydrogen bond is characterized by a BCP, a corresponding bond path, and a localized blue RDG isosurface, consistent with strong, CAHB. Furthermore, the QTAIM analysis identified two ancillary C–H···O hydrogen bonds, involving the carboxylate oxygen atoms that do not participate in the CAHBs. Due to the inherent ion-pair nature of the assembly, the calculated total formation energies for the tetramers are substantial: – 252.4 kcal/mol for **NOR–KET** and – 251.3 kcal/mol for **NOR–DKT**. To isolate the intrinsic strength of the interactions from these large, nondirectional Coulombic effects, the QTAIM energy predictor (E ≈ 0.5 × V) was applied. This analysis revealed very large stabilization energies for the individual N–H···O contacts forming the *R*_4_^2^(8) synthon: – 34.6 kcal/mol in **NOR–KET** and – 35.5 kcal/mol in **NOR–DKT**. Such high values confirm the crucial role and dominance of these CAHBs in dictating the solid-state structure of both salts. The secondary C–H···O contacts provide only minor stabilization, with energies of – 2.9 kcal/mol (**NOR–KET**) and – 2.8 kcal/mol (**NOR–DKT**). **NOR–NIF** exhibits a distinct trimeric assembly with a calculated total formation energy of – 125.7 kcal/mol. These trimeric units are the main source of union between the tetramers described in the crystal structure section. Here, the QTAIM analysis shows a bifurcated N–H···O,O hydrogen bond between the two cationic units, characterized by two BCPs, bond paths, and localized green RDG isosurfaces and established between the equatorial N–H bond and both O-atoms of the carboxylic group. The connectivity between the cation and anion is achieved through three BCPs and bond paths: one primary N–H···O CAHB, and two corresponding to bifurcated C–H,H···O contacts. The N–H···O interaction in **NOR–NIF** is very strong, evidenced by the intense blue color of its RDG isosurface. The estimated strength of all three NH···O contacts is – 25.2 kcal/mol, which is noticeably smaller than those found in **NOR–KET** and **NOR–DKT** tetramers. In contrast, the energetic contribution of the secondary C–H···O contacts in **NOR–NIF** trimer is very similar to the other salts, at – 2.6 kcal/mol.

**Figure 8:**
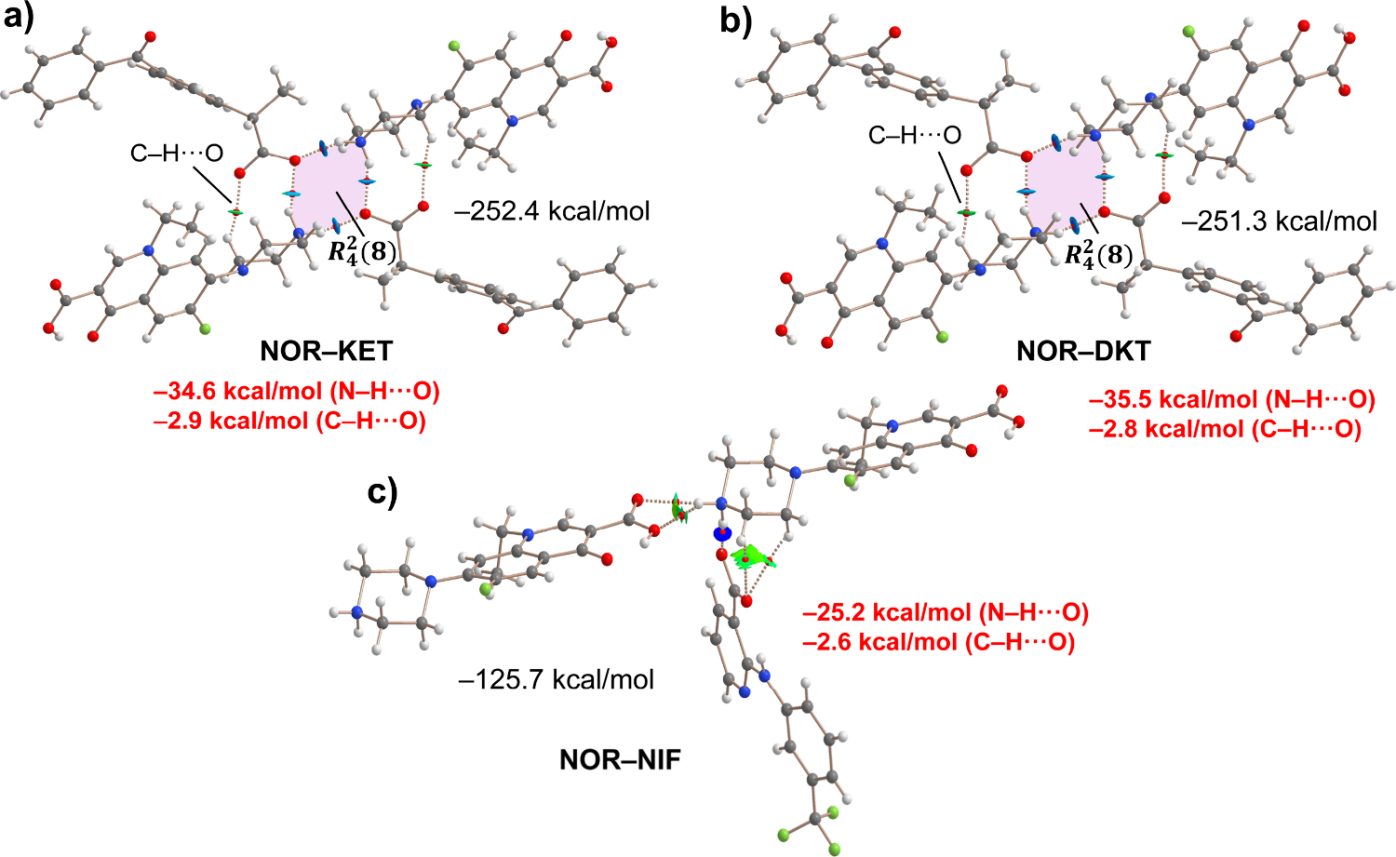
QTAIM and NCIplot analysis of the dominant assemblies in the pharmaceutical salts. a,b) *R*_4_^2^(8) synthon formed by the tetrameric unit observed in **NOR–KET** and **NOR–DKT**. c) Trimeric assembly in **NOR–NIF**. The interactions are characterized by BCPs (small red spheres) and Bond Paths (brown dashed lines). The RDG isosurfaces are mapped onto the sign of the second density Hessian eigenvalue (sign­(λ_2_)­ρ), where intense blue represents strong CAHBs and green represents weaker van der Waals interactions.

The hydrogen-bond networks observed in **NOR–KET** and **NOR–DKT c**onstitute the main source of stabilization. In contrast, although the tetrameric assemblies in **NOR–NIF** are propagated into chains through the N–H···O hydrogen-bonding interactions described above (trimeric assemblies), the association between adjacent chains is mainly governed by nonclassical noncovalent interactions (Figure 9). While only π···π interactions were described in the crystal-structure section, the computational analysis reveals that this stabilization also involves LP···π interactions, leading to a large formation energy of – 116.5 kcal/mol. This value is comparable to that calculated for the hydrogen-bonded trimeric assembly (Figure 8c), confirming the relevance of these contacts in controlling the final solid-state packing of **NOR–NIF**. DFT calculations also revealed CF_3_···π interactions that are topologically characterized by three BCPs and corresponding bond paths, interconnecting the three fluorine atoms of the CF_3_ group to the π–system of the quinoline ring (Figure 9). The NCIplot method further characterizes this interaction through localized green RDG isosurfaces, consistent with weak, dispersion-driven contacts. The central motif involves a strong π–stacking interaction between the two adjacent **NOR** ^**+**^ molecules. In this region, the green RDG isosurfaces extend over the entire space between the molecules, indicating a strong, stabilizing complementarity between the aromatic rings, supporting the crystal structure analysis. It is noteworthy that the stacking is not limited to the aromatic cores; the aliphatic piperazine ring of the **NOR** ^**+**^ unit is also stacked over the carboxylic group of the quinoline ring of the adjacent **NOR** ^**+**^ unit. This additional C–H···O interaction is topologically confirmed by the presence of three BCPs and bond paths connecting C–H groups of the piperazine ring to the oxygen atoms of both the carboxylic and quinone functionalities of the neighboring **NOR** ^**+**^ cation, thereby maximizing the total cohesive energy of the assembly.

**Figure 9:**
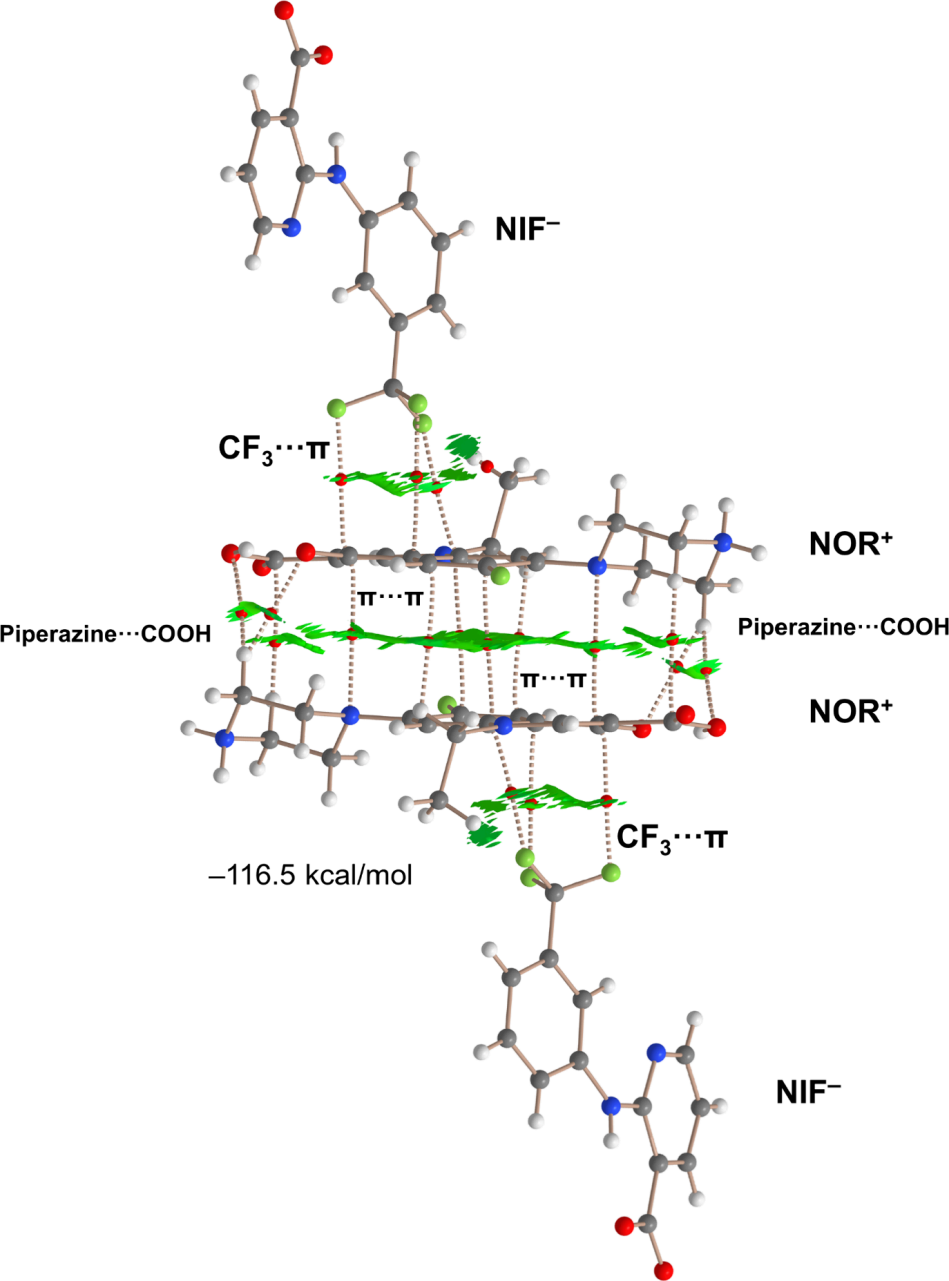
Topological analysis, using QTAIM and NCIplot method, of the noncovalent interactions present in the supramolecular assembly of **NOR–NIF** tetrameric units. BCPs are represented as small red spheres and Bond Paths as brown dashed lines. The RDG isosurfaces are mapped onto the sign of the second density Hessian eigenvalue (sign­(λ_2_)­ρ).

### 3.4 Stability Studies

Having established the supramolecular organization and quantified the interaction energetics, the next step is to evaluate how these structural features impact the physicochemical properties of the novel salts.

The thermal behavior was evaluated by simultaneous DSC–TGA analysis. As shown in Figure 10, the DSC thermograms display a single, well-defined endothermic event for each compound, corresponding to their melting point. The absence of additional thermal events in the DSC, along with the lack of mass loss before melting in the TGA traces (Figure S4), discards polymorphic transitions, or dehydration events before melting, while confirming phase purity.

**Figure 10:**
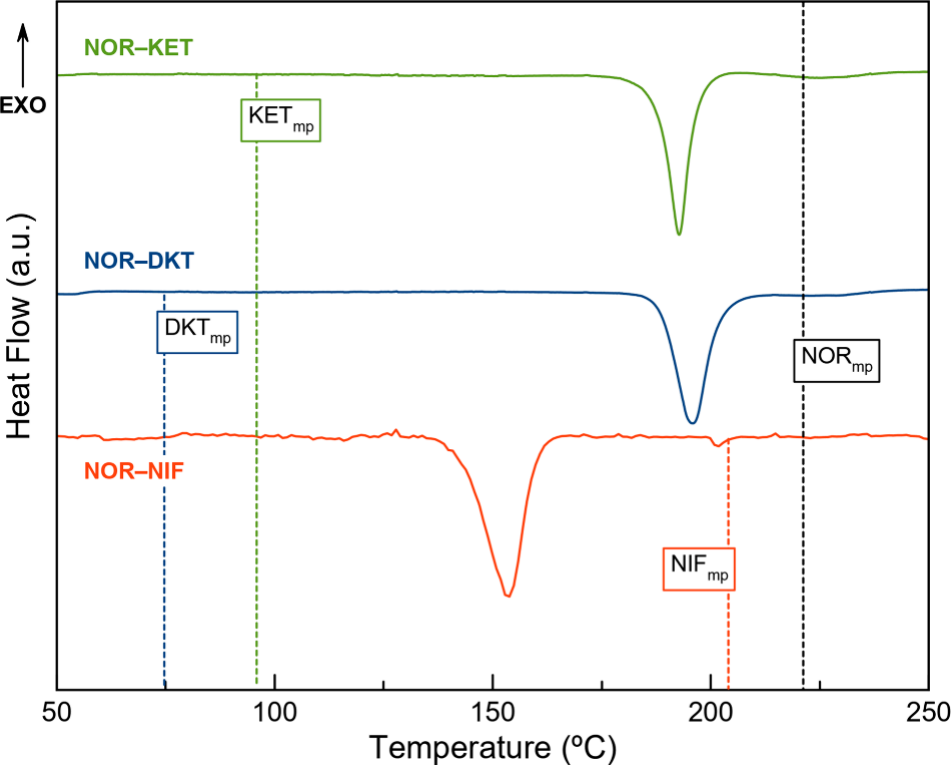
DSC traces of the **NOR–KET**, **NOR–DKT** and **NOR–NIF** along with the melting point of the APIs (dotted lines).

The melting point of the salts fall in between the melting point of pure **NOR** (221 °C) and the respective **NSAIDs**, which is a common behavior of PMMs.^57,58^ **NOR–KET** and **NOR–DKT** displayed significantly increased melting points (192.7 and 195.9 °C, respectively) relative to **KET** (75 °C) and **DKT** (96 °C). This enhancement correlates directly with the high stability of the *R*_4_^2^(8) tetrameric assembly identified in the salts, thus requiring higher thermal input to disrupt when compared with the dimeric association present in the native **NSAIDs.**

Interestingly, **NOR–NIF**exhibited the lowest melting point (153.6 °C), despite **NIF** presenting the higher melting point (203 °C) among the **NSAIDs**. Unlike the tetrameric organization in **NOR–KET** and **NOR–DKT**, **NOR–NIF** adopts a trimeric assembly with weaker CAHBs due to the presence of an intramolecular hydrogen bond in **NIF** ^–^, which delocalizes acceptor electron density and diminishes CAHB efficiency.

The stability of the salts under accelerated aging conditions was also evaluated by storing the samples at 40 °C and 75% RH for three months (Figure 11). Under these conditions, pure **NOR** transformed into its known sesquihydrate form (NOR·1.5H_2_O, CCDC 720746) in less than 24 h. In contrast, all three **NOR**–**NSAID** salts remained stable, retaining their original PXRD patterns and crystallinity. The strong hydrogen bonding between the **NSAIDs** and the protonated nitrogen atom in **NOR**’s piperazine ring blocked the hydration pathway, as the same nitrogen is involved in the coordination of water in **NOR** sesquihydrate.

**Figure 11:**
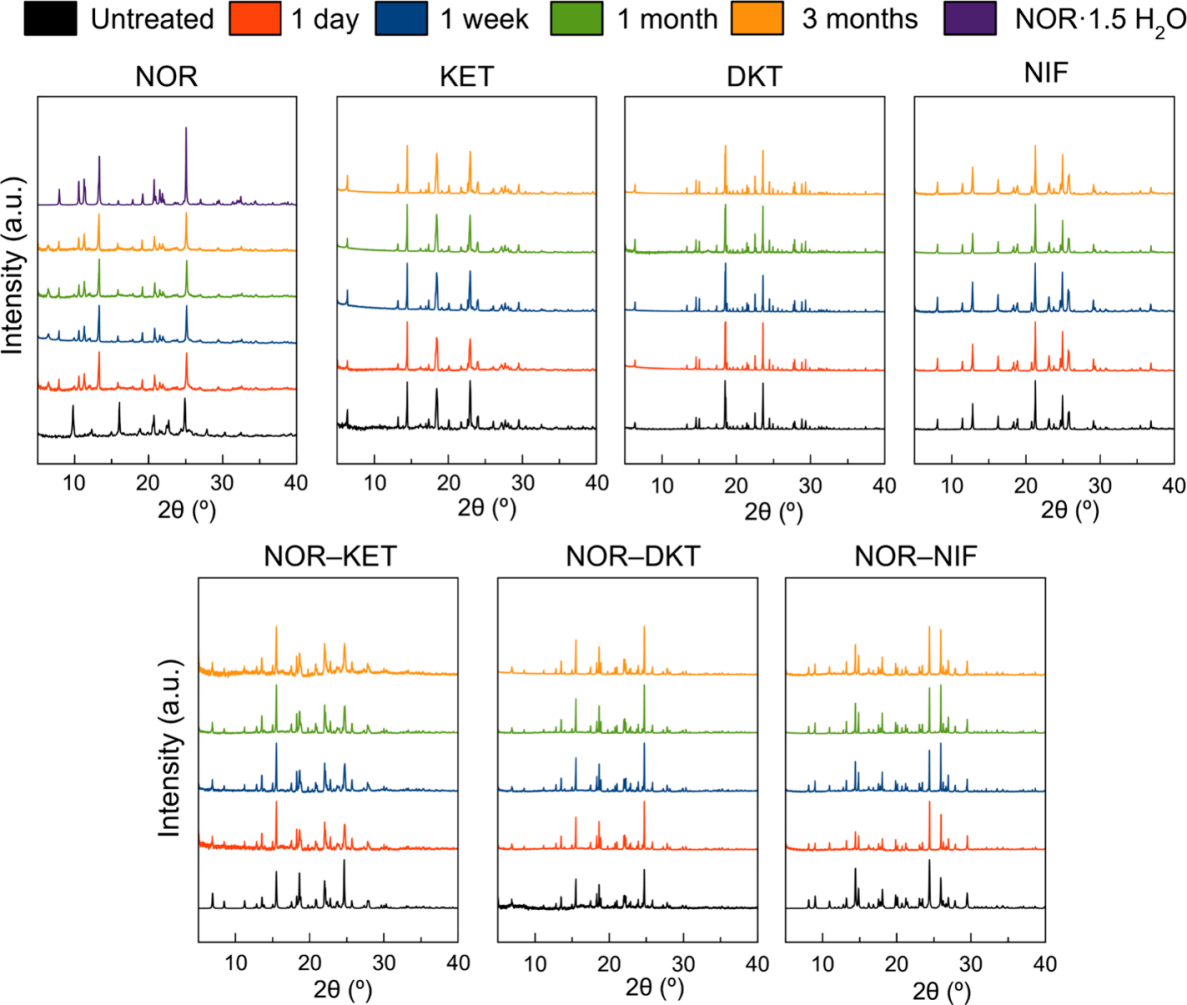
PXRD of the salts and the parent APIs under accelerated aging conditions. NOR·1.5H_2_O, CCDC number 720746, is also represented for comparison.

The thermodynamic stability in aqueous solution was assessed under two physiologically relevant conditions, KCl at pH 1.2 and PBS at pH 6.8, simulating the stomach and intestinal environments. As shown in Figure 12, at pH 1.2, **NSAIDs** remained stable under both pH conditions, whereas **NOR** transformed into the same unidentified phase observed during water-LAG, suggesting the formation of a **NOR** hydrate. In parallel, all salts underwent dissociation, with PXRD patterns revealing precipitated **NSAIDs**. This behavior is consistent with the amphoteric nature of **NOR**, which becomes protonated in acidic media, disrupting the salt and increasing **NOR** solubility while leaving the **NSAID** undissolved.

**Figure 12:**
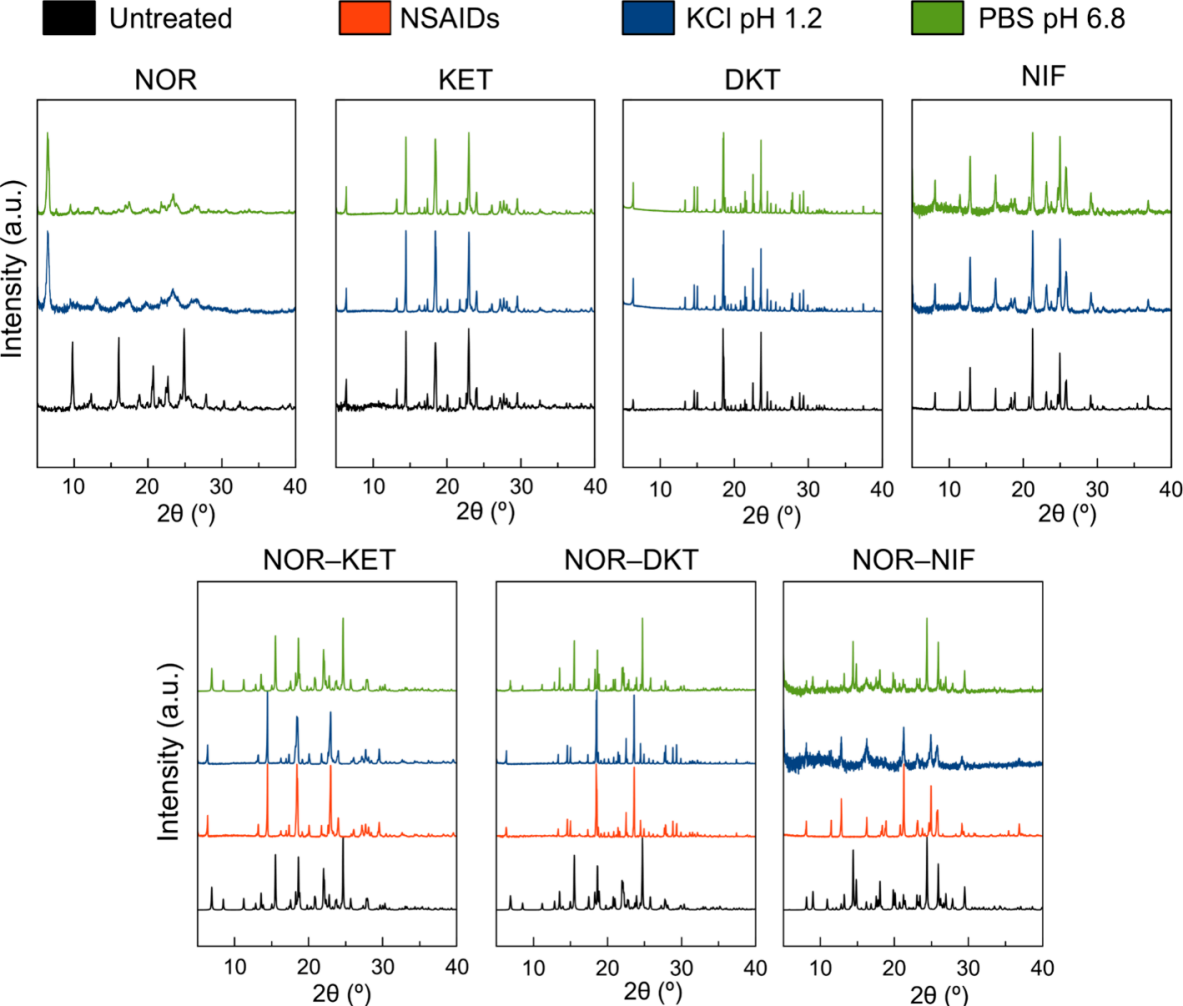
PXRD patterns of the parent APIs and the novel NOR–NSAID salts after 24 h in aqueous suspension in KCl at pH 1.2 and PBS at pH 6.8.

Despite the instability of the salts observed at acidic pH, this does not represent a pharmacological limitation, as both **NOR** and **NSAIDs** are primarily absorbed in the intestinal tract and gastroprotective formulations could be employed. At pH 6.8, **NOR**–**NSAID** salts remained chemically stable, with only **NOR–NIF** showing partial loss of crystallinity, which is a consequence of the trimeric assemblies that led to a less cohesive hydrogen bonding network, as demonstrated by the DFT calculations. These results demonstrated that salt formation with **NSAIDs** effectively protected **NOR** against hydration, not only under environmental humidity but also in aqueous solution.

### 3.5 Powder Dissolution Profile

The solubility behavior was evaluated under physiological conditions (PBS pH 6.8) and acidic medium (KCl, pH 1.2) (Figure 13). The good agreement between the reported solubility values of the APIs and those obtained in this work confirmed the reliability of the experimental data.^59–62^ In PBS, **NOR–KET** allowed a marked enhancement in the solubility of both components, reaching concentrations of 0.96 mg/mL for **NOR** and 0.89 mg/mL for **KET** within the first 50 min, followed by a sustained increase over 24 h, reaching equilibrium concentrations of 1.42 mg/mL (**NOR**) and 1.20 mg/mL (**KET**). In the same conditions, native **NOR** showed a 5-fold lower equilibrium solubility (0.294 mg/mL) along with a less stable and more erratic supersaturation profile in the first minutes. Pure **KET** also displayed very limited solubility (∼0.2 mg/mL), reaching equilibrium within the first minutes of the experiment. A similar trend was observed for **NOR–DKT**, consistent with the structural similarity between both salts. The system reached equilibrium solubilities of 1.52 mg/mL for **NOR** (x5 increase) and 1.12 mg/mL for **DKT** (x4 increase).

**Figure 13:**
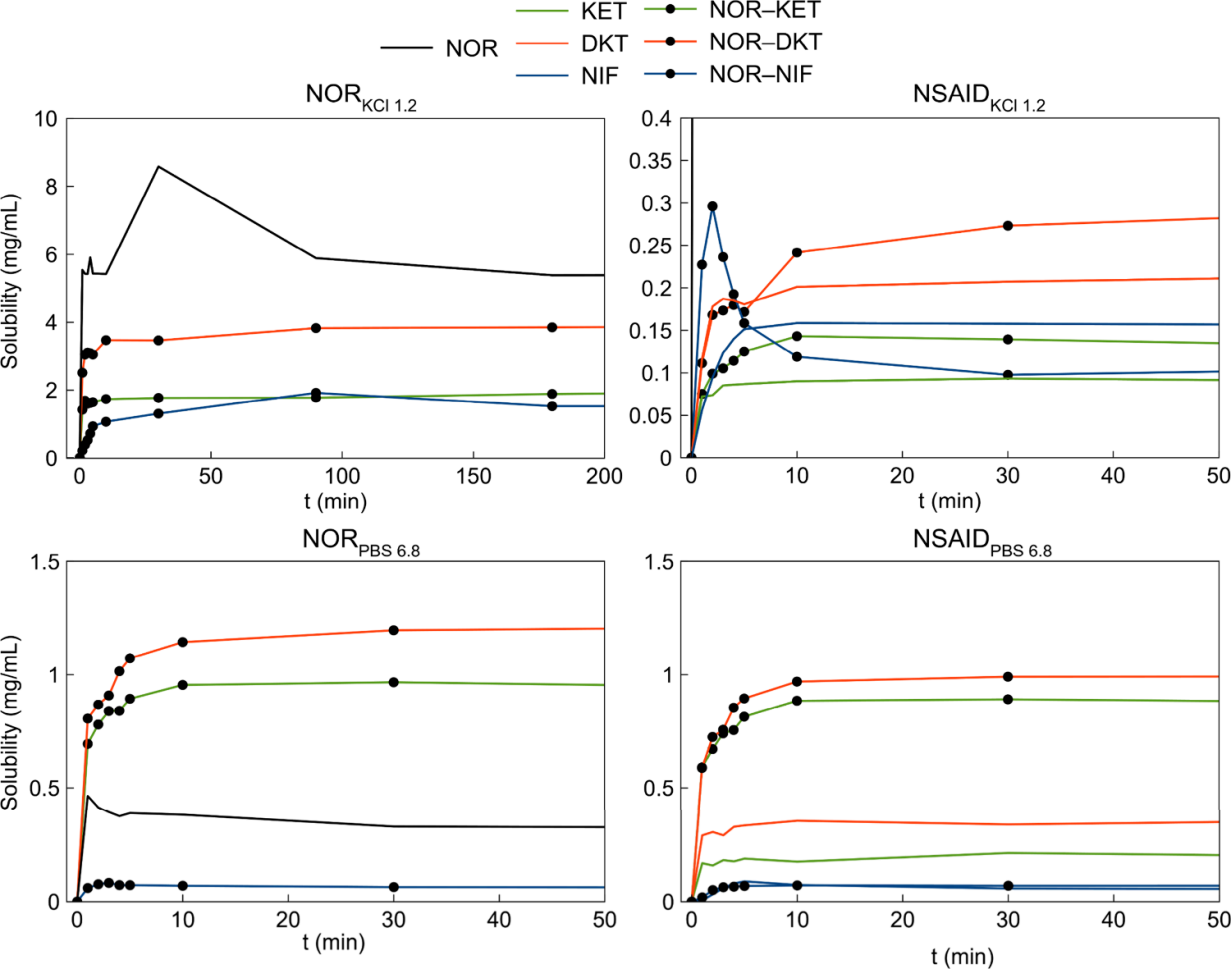
Solubility profiles of the APIs and the salts at KCl pH 1.2 and PBS pH 6.8.

In contrast, **NOR–NIF** achieved 0.068 mg/mL for **NOR** and 0.089 mg/mL for **NIF**. This downregulation (x4) is consistent with the intrinsically poor solubility of **NIF** (∼0.02 mg/mL), which limits the water uptake in the crystal lattice and the dissolution of the salt. No pronounced spring–parachute phenomenon was observed for **NOR–NIF**, despite **NOR** and **NIF** presenting supersaturation phenomena in the early stages.

In KCl pH 1.2, as demonstrated in the stability section, the salts underwent dissociation, thus the solubility of the **NSAIDs** remained comparable to that of the native drugs (∼0.09–0.10 mg/mL). Native **NOR** displayed very high solubility, reaching an initial supersaturation peak of 8.58 mg/mL and stabilizing at approximately 9.40 mg/mL. Interestingly, all salts exhibited a more controlled dissolution profile for **NOR**, with no supersaturating effects, lower initial concentrations and a gradual increase over time. For **NOR–KET**, concentrations increased from ∼ 2 mg/mL to ∼ 5 mg/mL over 48 h, while **NOR–DKT** showed a faster initial release (∼4 mg/mL) followed by a slower increase toward similar equilibrium values. Salt formation and further dissociation in acid media allow the salts to act as reservoirs that modulate **NOR** release and the final equilibrium concentrations of **NOR**. Notably, **NOR–NIF** led to the lowest solubility of **NOR** (∼2 mg/mL). In this case, the structure is dominated by hydrophobic π···π stacking and CF_3_···π interactions with a reduced contribution from CAHBs, which decrease the affinity of the affinity of the system for aqueous molecules, leading to reduced dissolution. These results are particularly relevant from a pharmacological perspective, as controlling the high solubility and supersaturation of **NOR** at acidic pH, where absorption is minimal, may reduce local accumulation and associated side effects.

## 4 Conclusions

This work demonstrates that drug–drug salt formation between **NOR** and **NSAIDs** is a powerful strategy to simultaneously tune stability, solubility, and release behavior. Structural and computational analyses revealed that robust CAHB-stabilized tetrameric architectures in **NOR–KET** and **NOR–DKT** lead to highly stable crystal packings, whereas π-stacking and CF_3_···π interactions dominate the structure of **NOR–NIF**, resulting in weaker cohesion.

The structural differences directly translate into pharmaceutical performance. The cohesive tetrameric structures of **NOR–KET** and **NOR–DKT** significantly improved the thermal stability of the **NSAIDs**, approximately doubling their melting points, whereas the less cohesive **NOR–NIF** structure led to less thermal stability. Additionally, salt formation suppressed **NOR** hydration by blocking its primary water coordination site, enhancing the stability under both accelerated aging and aqueous conditions. At the same time, the salts modulated the dissolution behavior, enhancing NOR solubility under physiological conditions while reducing excessive supersaturation in acidic media through a reservoir-like release mechanism. Interestingly, the lower number of CAHBs combined with the greater contribution of hydrophobic π–π interactions in **NOR–NIF** resulted in a less water-accessible structure and reduced **NOR** solubility.

Overall, this study highlights how rational crystal engineering enables direct control of macroscopic drug performance. Beyond combining therapeutic functionalities, this approach provides a robust framework for designing solid forms with optimized stability, bioavailability, and controlled release, establishing a solid foundation for future pharmaceutical development.

## Supplementary Material



## Notes

Supplementary table and figures comprehensively document: (1) PXRD patterns of products obtained by neat grinding and LAG in H_2_O, along with the PXRD of the parent APIs; (2) PXRD patterns of the products obtained after 1:1 reaction of NOR and NIF in a) LAG using different organic solvents, and b) slurry reactions in MET at different times; PXRD patterns of the parent APIs are also included for better comparison; (3) FT-IR spectra of the pure bulk phases obtained from LAG (NOR–KET and NOR–DKT) and slurry reactions (NOR–NIF) compared with the parent APIs (PDF)
