# Supplementary material for: Norfloxacin–NSAID Pharmaceutical Salts: Structure–Property Relationships for Enhanced Solubility and Stability
Source: Cryst Growth Des. 2026 Jun 11;26(13):5204–17. doi: 10.1021/acs.cgd.6c00593 (PMC13335406; doi:10.1021/acs.cgd.6c00593)
Supplement: Supplementary file 1 [file cg6c00593_si_001.pdf]

Norfloxacin–NSAID Pharmaceutical Salts: Structure–Property Relationships for Enhanced Solubility and Stability.

Jeannette Carolina Belmont-Sánchez,[1,2] *Carolina Alarcón-Payer,*[3] *Antonio Frontera,*[4] *Francisco Javier Acebedo-Martínez,*[5,*] *Cristóbal Verdugo-Escamilla,*[1] *Alicia Domínguez-Martín,*[2] *and Duane Choquesillo-Lazarte*[*1]

[1] Laboratorio de Estudios Cristalográficos, IACT, CSIC, Avda. de las Palmeras 4, 18100 Armilla, Spain.

[2] Department of Inorganic Chemistry, Faculty of Pharmacy, University of Granada, 18071 Granada, Spain.

[3] Servicio de Farmacia, Hospital Universitario Virgen de las Nieves, 18014 Granada, Spain.

[4] Departament de Química, Universitat de les Illes Balears, Crta. de Valldemossa km 7.5, 07122 Palma, Spain.

[5] Istituto per lo Studio dei Materiali Nanostrutturati (ISMN)-Consiglio Nazionale Delle Ricerche (CNR), Via P. Gobetti 101, 40129 Bologna, Italy.

Electronic Supplementary Information

Table of contents

Figure S1. PXRD patterns of products obtained by neat grinding and LAG in H2O, along with the PXRD of the parent APIs.

Figure S2. PXRD patterns of the products obtained after 1:1 reaction of NOR and NIF in a) LAG using different organic solvents, and b) slurry reactions in MET at different times. PXRD patterns of the parent APIs are also included for better comparison.

Figure S3. FT-IR spectra of the pure bulk phases obtained from LAG (**NOR–KET** and **NOR–DKT)** and slurry reactions (**NOR–NIF)** compared with the parent APIs.

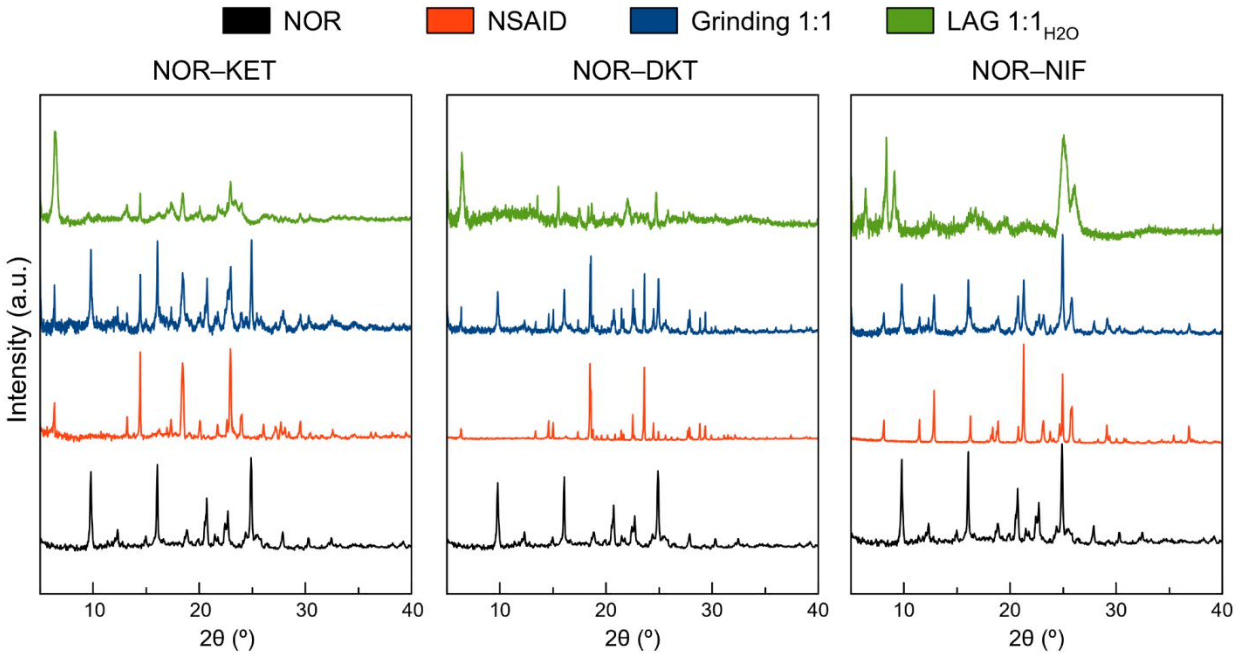

Figure S1. PXRD patterns of products obtained by neat grinding and LAG in H_2O, along with the PXRD of the parent APIs.

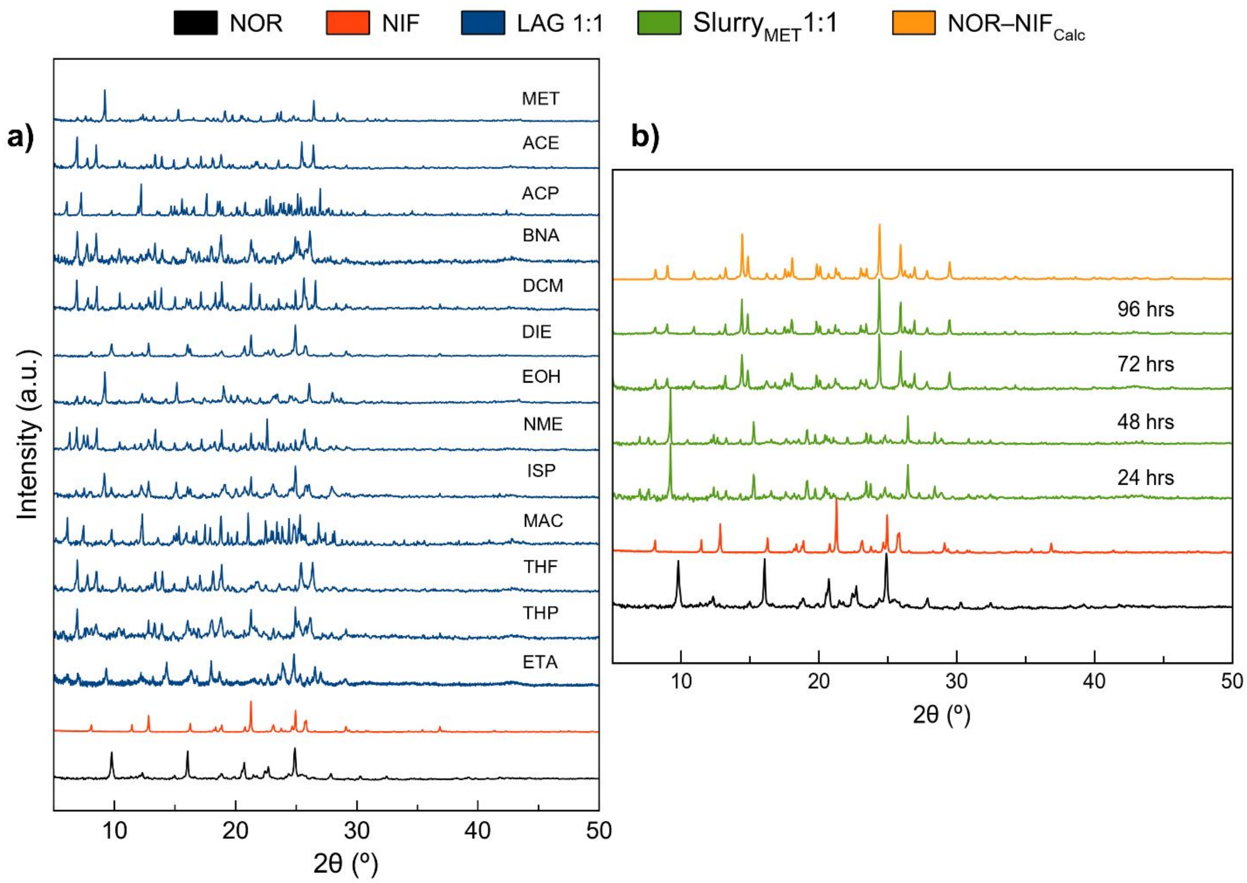

Figure S2. PXRD patterns of the products obtained after 1:1 reaction of NOR and NIF in a) LAG using different organic solvents, and b) slurry reactions in MET at different times. PXRD patterns of the parent APIs are also included for better comparison.

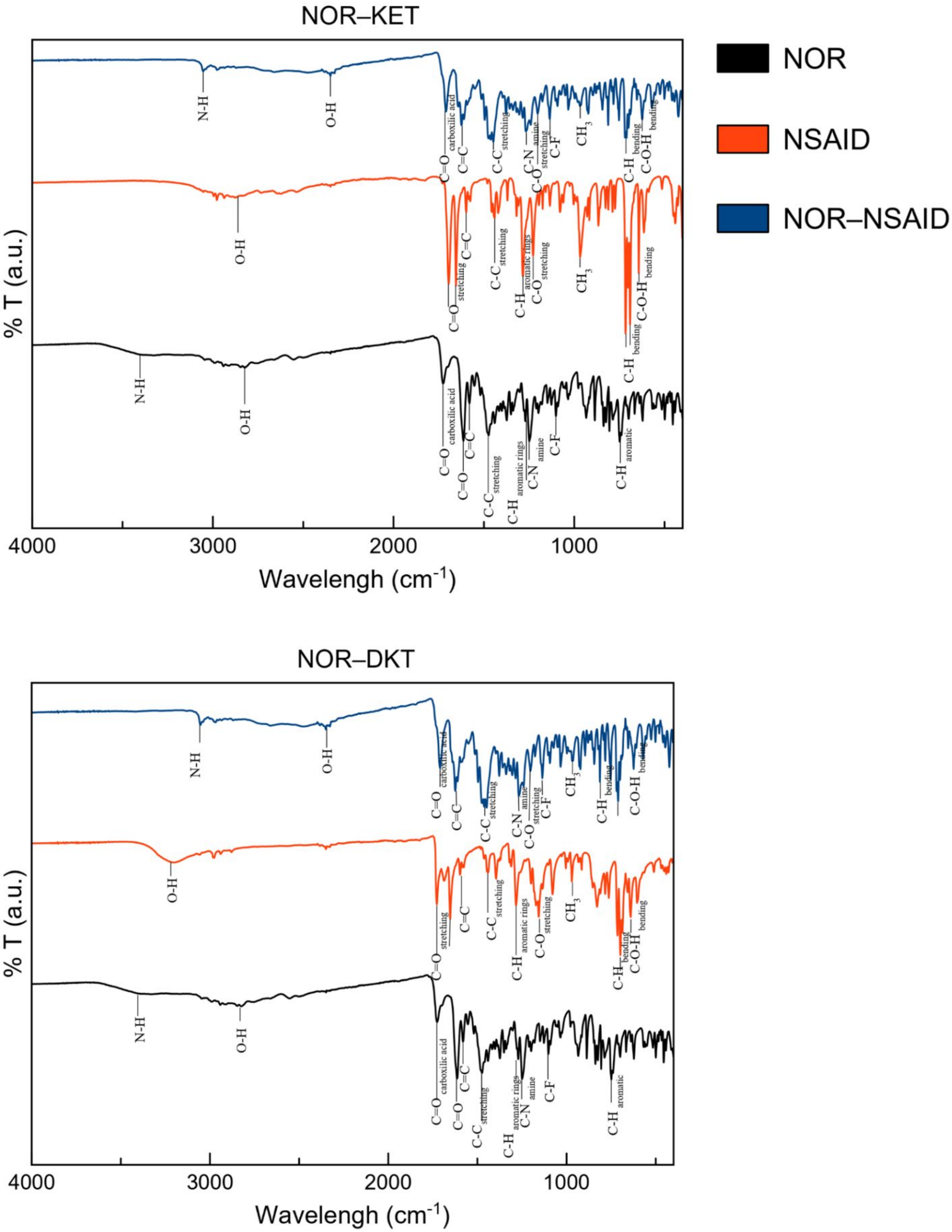

NOR–KET
NOR
NSAID
NOR–NSAID
% T (a.u.)
Wavelengh (cm-1)
4000
3000
2000
1000
N-H
O-H
C=O
carboxilic acid
C=C
C-C stretching
C-N amine
C-O stretching
C-F
CH3
C-H bending
C-O-H bending
C=O stretching
C-H aromatic rings
C-H aromatic
NOR–DKT

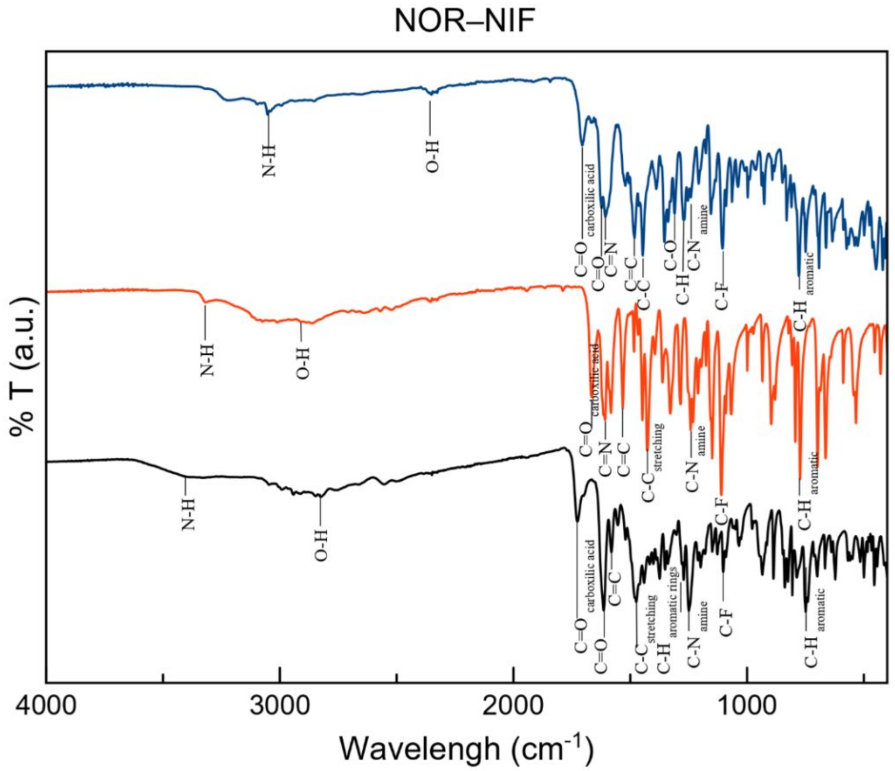

Figure S3. FT-IR spectra of the pure bulk phases obtained from LAG (**NOR–KET** and **NOR–DKT)** and slurry reactions (**NOR–NIF)** compared with the parent APIs.

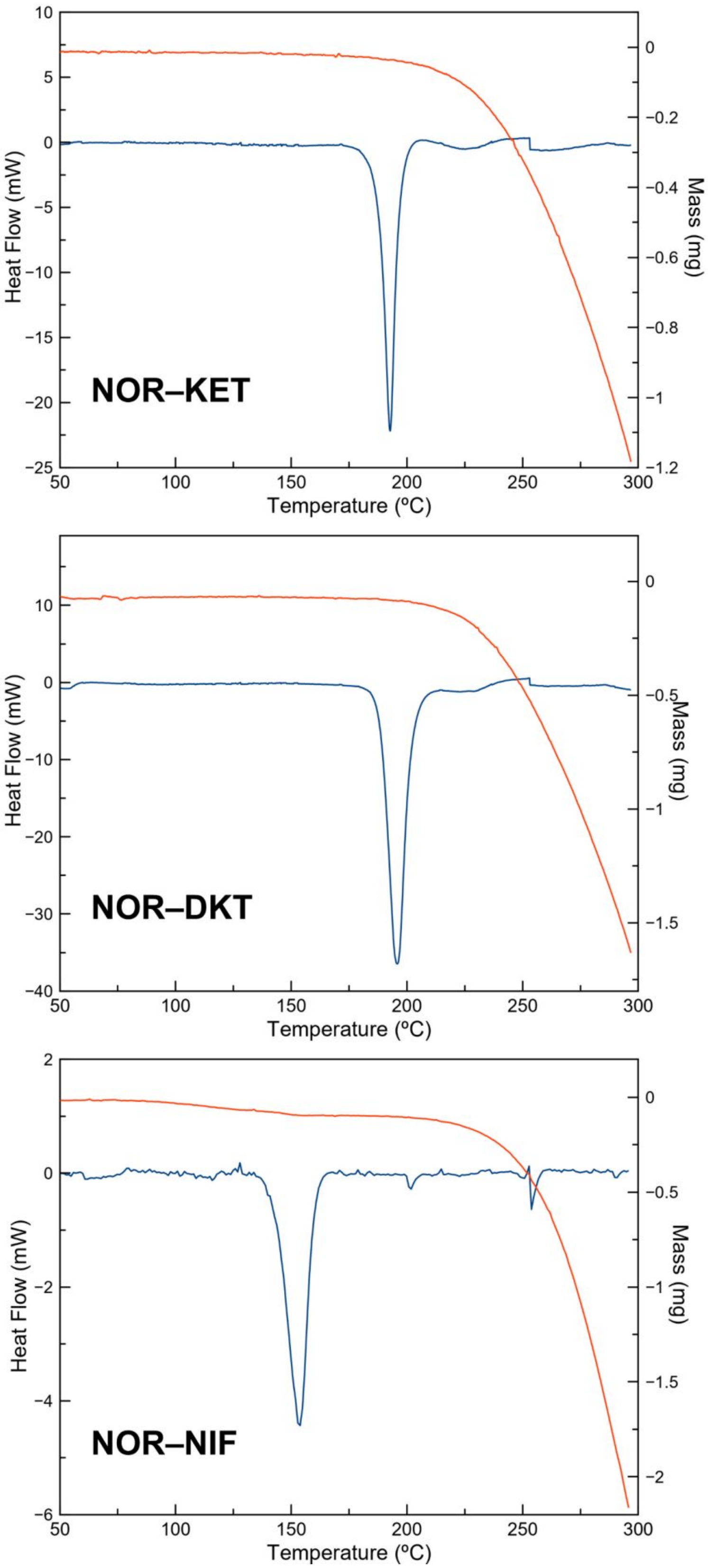

NOR–KET
NOR–DKT
NOR–NIF
Heat Flow (mW)
Mass (mg)
Temperature (ºC)

Figure S4. DSC (Blue) and TGA (Red) traces of the **NOR–KET**, **NOR–DKT** and **NOR–NIF**.
